# Alternative Approaches and Plant‐Based Remedies for Livestock Health Management Among the Batswana of Southern Africa: A Review

**DOI:** 10.1002/cbdv.202503248

**Published:** 2026-04-17

**Authors:** Tswelelopele G. Mpolokeng, Ndzalama Shikwambana, Mompati V. Chakale, John A. Asong, Lyndy J. McGaw, Stephen O. Amoo, Nqobile A. Masondo, Adeyemi O. Aremu

**Affiliations:** ^1^ Indigenous Knowledge Systems Centre Faculty of Natural and Agricultural Sciences North‐West University Mmabatho South Africa; ^2^ South African Research Chairs Initiative in Indigenous Knowledge‐Driven Medicinal Plants Utilisation and Conservation Strategies for Human, Animal, and Crop Health (IK‐Medplants4HAC), Faculty of Natural and Agricultural Sciences North‐West University Mmabatho South Africa; ^3^ Agricultural Research Council – Vegetable, Industrial and Medicinal Plants Pretoria South Africa; ^4^ Phytomedicine Programme, Department of Paraclinical Sciences Faculty of Veterinary Science University of Pretoria Onderstepoort South Africa; ^5^ Unit For Environmental Sciences and Management, Faculty of Natural and Agriculture Sciences North‐West University Potchefstroom South Africa; ^6^ School of Agriculture and Science College of Agriculture Engineering and Science University of KwaZulu‐Natal Durban South Africa

**Keywords:** animal health, antimicrobial, bioactivity, one health, phytochemicals

## Abstract

Due to limited access to, and the high cost of conventional veterinary services, Batswana communities often rely on ethnoveterinary practices for livestock health management. This review provides an in‐depth analysis on the ethnoveterinary uses, biological properties and safety assessment of plants utilised in livestock husbandry. A systematic literature search was conducted using scientific databases, focusing on articles published from 1997 to 2024. After generating the inventory of plants with ethnoveterinary data, further search was conducted to assess the documented biological activities, safety, and phytochemicals for the recorded plants. A total of 116 plants were documented as remedies for managing nine livestock conditions. The most cited health conditions were retained placenta (81 citations), diarrhoea (65), and wounds (44). The most prominent plants were *Senna italica* (10 citations), *Terminalia sericea* (8 citations), and *Ziziphus mucronata* (8 citations). Approximately 52% of the 116 plants with ethnoveterinary records have empirical data on their biological effect, safety, and phytochemicals. Antimicrobial screening was the most common assay conducted (36%), which dominantly used microbial strains such as *Staphylococcus* spp., *Pseudomonas aeruginosa*, and *Escherichia coli*. We established the vital role of ethnoveterinary practices in Batswana livestock management and the potential of plants in sustainable veterinary care.

## Graphical abstract

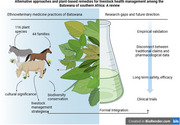

**Abbreviations**
CC: column chromatography
DEPT: distortionless enhancement by polarisation transfer
EI‐MS: electron ionisation‐mass spectrometry
FTIR: Fourier transform infrared
GC‐MS: gas chromatography‐mass spectrometry
HPLC: high performance liquid chromatography
IR: infrared
LC‐MS: liquid chromatography‐mass spectrometry
NMR: nuclear magnetic resonance
TLC: thin layer chromatography
UHPLC‐qTOF‐MA:  ultra‐high performance liquid chromatography‐quadrupole time‐of‐flight mass spectrometer
UPLC: ultra performance liquid chromatography
UV: ultraviolet

## 1 Introduction

Globally, livestock such as cattle, goats, sheep, chickens, and horses play crucial roles in human life by providing food, generating income, and supplying materials, while also symbolising wealth, and are linked to social standing and cultural heritage. Additionally, they contribute to tourism and employment opportunities [1, 2, 3]. Livestock husbandry is an integral part of the livelihoods of Batswana communities in southern Africa, providing economic support and cultural significance [4, 5]. The Batswana are part of the Bantu‐speaking people and found across several countries in southern Africa [6, 7]. Archaeological evidence suggests that livestock rearing took place from the later Stone Age in southern Africa [8], with historical link to east Africa [9]. Livestock have always been significant to the Bantu‐speaking agropastoral people of southern Africa, and still in present times, they remain important commodities used for wealth transfer and are valued in some cultures for their connections with ancestors [10]. The Batswana were selected as the focus of this review due to their wide geographical distribution across southern Africa, strong livestock‐based livelihoods, and well‐documented reliance on ethnoveterinary medicine [11]. Despite this, existing knowledge remains fragmented, necessitating a consolidated and critical synthesis. Livestock contribute greatly to food security in rural communities, provide invaluable ecological services, and are also used in traditional rituals [12].

However, limited access to conventional veterinary services, coupled with their high cost, has led to the widespread reliance on traditional methods for managing livestock health [13]. These methods, deeply rooted in indigenous knowledge systems, often involve the use of plant‐based remedies to manage a variety of livestock ailments [14]. Plant‐based remedies have long been recognised as an affordable and sustainable alternative to conventional veterinary medicine [15]. Among the Batswana, these remedies are employed to manage conditions ranging from reproductive disorders and gastrointestinal problems to respiratory infections and wounds.

Veterinary phytomedicine has long been practiced by indigenous communities worldwide. In sub‐Saharan Africa, its effectiveness has largely been based on oral traditions and practical use rather than formal documentation [16]. In contrast, other regions such as India have preserved records of traditional veterinary medicine in Ayurvedic texts [17]. These remedies are believed to have developed through trial and error or by observing animal self‐medication [17]. Many medicinal plants used in traditional veterinary practices contain bioactive compounds with antimicrobial, antioxidant, anti‐inflammatory, and anti‐parasitic properties, making them valuable for treating infections, wounds, and other livestock health issues [18]. Southern Africa, which is recognised as a biodiversity hotspot, harbors numerous plant species with potential for veterinary applications [19, 20]. The secondary metabolites in these plants contribute to animal health and provide a cost‐effective alternative to synthetic drugs. They also help address critical challenges such as antimicrobial resistance and drug residues in animal products [21, 22]. Despite the widespread use and cultural significance of these remedies, scientific documentation and validation/valorisation of their efficacy remain sparse [23, 24].

Furthermore, understanding the pharmacological properties of these plants presents an opportunity to develop affordable and accessible veterinary products with proven efficacy and safety [25]. The current review entails an appraisal of the existing ethnoveterinary knowledge, biological activities, and phytochemical profile of plants used for managing livestock health among the Batswana in southern Africa. By highlighting the strengths and gaps in the current knowledge, the review aims to contribute to the increasing body of evidence supporting sustainable livestock management practices in southern Africa. Additionally, it identifies opportunities for future research into the pharmacological potential of traditional remedies, emphasising the importance of preserving, valorising, and integrating indigenous knowledge into contemporary veterinary medicine.

## 2 Methods

The review is based on published ethnoveterinary studies conducted amongst Batswana communities from January 1997 to June 2024. The systematic review is structured according to PRISMA guidelines [26]. Electronic databases such as Google Scholar, ScienceDirect, and Scopus were used to search for literature. Furthermore, published literature from dissertations, theses, and ethnobotanical books retrieved from the North‐West University online repositories were used in the review. Diverse keywords and phrases were used to access eligible articles. These included “medicinal plants for livestock, livestock health management, Batswana, indigenous knowledge, livestock management, southern Africa, and ethnoveterinary practices”. The Boolean operators of ‘AND’ and ‘OR’ were included to extend the search. Bibliographies of selected articles were also examined to identify further references that might have been omitted from the initial searches. The articles included in this review focused on Batswana communities in southern Africa and explicitly reported the use of ethnoveterinary medicine in managing livestock health care. The collected information included Latin and local names of the plants, plant parts, diseases or conditions treated, preparation methods and mode of administration, and the classification of livestock conditions. Publications were excluded if they focused on modern or non‐plant‐based veterinary practices, were conducted outside southern Africa, did not focus on Batswana communities, or lacked sufficient details on the ethnoveterinary practices and plant species used. Studies not available in English were also excluded. All scientific plant names were verified using the “Plants of the World Online | Kew Science” (https://powo.science.kew.org/).

**FIGURE 1:**
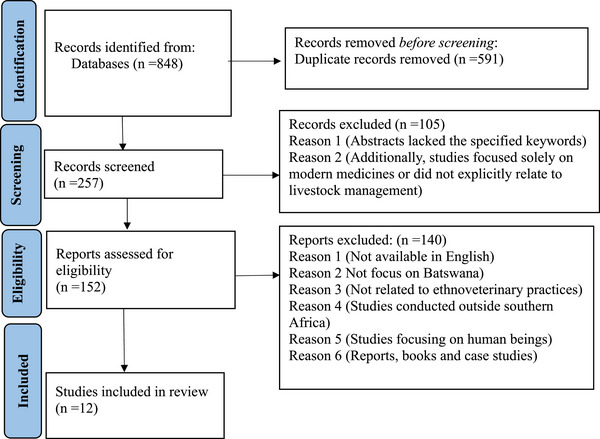
Flow diagram for selection of articles used for generating inventory of ethnobotanical practices and plant‐based remedies for managing livestock health conditions among the Batswana in southern Africa.

A total of 848 studies were recorded from various scientific databases (Figure 1), which included journal articles, theses, books, and dissertations on the ethnoveterinary studies conducted across southern Africa from 1997 until June 2024. During the screening phase, the titles and abstracts of all the articles were reviewed. A total of 591 duplicate articles were removed after applying the eligibility criteria. Following an additional individual screening of the remaining 257 studies, 105 articles were removed because their abstracts lacked the specified keywords, the studies focused solely on modern medicines, or did not explicitly relate to livestock management. A total of 140 articles were also excluded either because they are not available in English language, did not focus on Batswana, were not related to ethnoveterinary practices, such as those on modern or non‐plant‐based veterinary methods or the studies were conducted outside southern Africa, while the remaining 12 studies were eligible (Figure 1).

To assess the biological activity and safety assessment as well as the phytochemical profiles of plants identified as being used in Batswana ethnoveterinary medicine, a further literature search guided by the generated plant inventory and using the target biological activities as search keywords was undertaken. Journals, books and reports that focused on animal health were considered. The literature was searched using specific keywords on international databases such as Scopus, Web of Science and ScienceDirect.

## 3 Results and Discussion

### 3.1 Literature Search Output

In this review, the eligible studies covered two countries namely South Africa and Botswana. In terms of geographical distribution of the eligible ethnoveterinary studies, there were more studies among the Batswana communities in Botswana (58.33%) compared to eligible studies in South Africa (41.67%) (Table 1). Following a detailed analysis, 72.61% of the documented plants were from studies conducted in South Africa while 27.38% of the plants were from Botswana. Even though more studies were conducted in Botswana, the ethnoveterinary practices in South Africa contributed a higher portion in terms of diversity of plant species used for livestock health conditions. This could be attributed to several factors, such as ecological diversity in South Africa contributing to a broader range of medicinal plants, or more comprehensive documentation of plant species within South Africa [27].

**TABLE 1:** Overview of reviewed literature on ethnoveterinary plants used in livestock management by the Batswana in southern Africa.

Author(s)	Title of the study	Country	No. of plants	No. of families	Voucher specimen deposited?	Participants	Livestock treated	Methodological framework
Chakale et al. [76]	Ethnoveterinary practices and ethnobotanical knowledge on plants used against cattle diseases among two communities in South Africa	South Africa	64	32	Yes	Community members	Cattle	Semi‐structured interview, field walk
Gabalebatse et al. [56]	Ethnoveterinary practices amongst livestock farmers in Ngamiland District, Botswana	Botswana	11	9	Unspecified	Farmers or cattle herders	Cattle	Structured questionnaires
Gabanakgosi et al. [131]	Ethnoveterinary medicine usage in family chickens in the selected four villages of Botswana	Botswana	6	6	Unspecified	Farmers	Chicken	Structured questionnaires
Getchell et al. [109]	Raising livestock in resource‐poor communities of the North West province of South Africa‐a participatory rural appraisal study	South Africa	10	8	Unspecified	Farmers	Cattle, sheep, goats and chicken	Questionnaire and focus group
Lechani et al. [53]	Participatory inventory of plant‐based ethnoveterinary medicine used to control internal parasites of goats in the Ngamiland region of Botswana	Botswana	13	11	Unspecified	Communal farmers	Goats	Structured questionnaires
Moichwanetse et al. [52]	Ethnoveterinary plants used for the treatment of retained placenta and associated diseases in cattle among Dinokana communities, North West Province, South Africa	South Africa	25	18	Yes	Farmers and herders	Cattle	Semi‐structured interview
Moreki et al. [62]	Potential use of ethnoveterinary medicine for retained placenta in cattle in Mogonono, Botswana	Botswana	14	14	Unspecified	Herd boys and stockowners	Cattle	Rapid Rural Techniques (RRA)
Moreki [171]	Small‐scale poultry production systems in Serowe‐Palapye sub‐district	Botswana	5	3	Unspecified	Poultry farmers	Poultry	Interviews, focus group, direct observation conference and a seminar
Ndou et al. [64]	Indigenous knowledge and use of medicinal plants for ethnoveterinary within the North West Province, South Africa	South Africa	31	14	Yes	Farmers, traditional healer, community members	Cattle, sheep, goats and chicken	Semi‐structured interview
Setlalekgomo and Setlalekgomo [63]	The use of ethnoveterinary medicine in goats in Lentsweletau village in Kweneng District of Botswana	Botswana	13	12	Unspecified	Farmers	Goats	Structured questionnaires
Setlalekgomo [55]	Snakebite management in cattle by farmers in Lentsweletau extension area of Kweneng District in Botswana	Botswana	4	4	Unspecified	Farmers and cattle herders	Cattle	Structured questionnaires
Van der Merwe et al. [91]	Use of ethnoveterinary medicinal plants in cattle by Setswana‐speaking people in the Madikwe area of the North West Province of South Africa	South Africa	45	24	Unspecified	Farmers, extension officers, traditional healers, Knowledge holders	Cattle	Rapid Rural Techniques (RRA), group interviews, observation and field walk

The types of participants involved in each study has significant impact on the scope and depth of generated data [28]. Farmers (63.63%), community members (18.18%), traditional healers (18.18%), extension officers (9.09%), and knowledgeable elders (9.09%) provide first‐hand knowledge of plant usage in livestock health (Table 1). The expertise of farmers, community members, extension officers, knowledgeable elders, and traditional healers is largely derived from years of experience in livestock management, where they use traditional practices to address various health issues [29]. Such knowledge brings unique perspectives and practices to the preservation and application of indigenous health systems in managing livestock health. In many parts of Africa, these knowledge sources provide practical and accessible solutions for livestock health, serving as vital resources where modern veterinary services are lacking or not accessible [28].

Semi‐structured questionnaires (50%), rapid Rural Techniques (20%) and participatory research model (10%) were used to document the methodological framework of the studies in South Africa and Botswana (Table 1). The combination of semi‐structured questionnaires, rapid rural Appraisal (RRA) techniques, and participatory research approaches offers a comprehensive approach to studying ethnoveterinary practices [30, 31, 32]. These methods enable researchers to gather in‐depth, reliable data while fostering collaboration and inclusivity with Batswana communities [33]. The different methods provide an excellent opportunity to explore and experiment with various techniques, facilitating the collection of both qualitative and quantitative data [34]. The dual approach allows researchers to address contemporary theoretical issues surrounding the development, nature, and transmission of ethnobotanical knowledge [35].

### 3.2 Ethnoveterinary Status of Plant Species Used by the Batswana to Manage Livestock Health Conditions

Using the eligible literature, an analysis on the ethnoveterinary research that focused on the Batswana was conducted. Diverse aspect related to the identified plants and associated indigenous knowledge and practices are elaborated accordingly.

#### 3.2.1 Diversity of Plant Species With Ethnoveterinary Records

A total of 116 plant species from 44 families were recorded as being used in the management of livestock health conditions in South Africa and Botswana (Table 2). *Senna italica*, *Terminalia sericea*, *Ziziphus mucronata*, *Peltophorum africanum*, *Drimia sanguinea*, and *Aloe ferox* were the most cited plant species, representing 37.06% of the generated plant inventory. The plants are reported to be used as multifunctional medicine for the treatment of various livestock diseases, including gastrointestinal infections, respiratory disorders, wound healing, and ectoparasitic infestations. These conditions are among the most frequently cited in ethnoveterinary studies, highlighting the broad‐spectrum use of these plant‐based remedies. Additionally, the health benefits of some of the most cited ethnoveterinary plant species has been demonstrated in other African countries such as Cameroon [36], Namibia [37, 38], Ethiopia [39, 40], and Zimbabwe [41]. The prevalence of *Aloe* species in the disease management of Zimbabwean poultry (e.g., wounds, diarrhoea, and ectoparasites) was held to be indicative of efficacy for the plant [42]. Geographical distribution, availability and health benefits of *Aloe* species (*Aloe ferox*, *Aloe greatheadii*, *Aloe marlothii*, *Aloe vera*, *Aloe zebrina*) in different African regions could be the contributing factor in their common usage for disease management or conditions such as wounds, constipation and retained placenta. The dominance of the *Aloe* genus illustrates its pharmacological potential, adaptability and broad‐spectrum efficacy as the plants are frequently praised for their anti‐inflammatory, and laxative properties [43]. The patterns of findings on species such as *Aloe* sp. align with the patterns observed in South Africa and Botswana, suggesting a shared reliance on specific taxa across different regions of Africa. The relatively high citation frequency of other commonly used plants such as *Senna italica* and *Terminalia sericea* demonstrates their perceived effectiveness and suggests their broad applicability in managing livestock conditions. In terms of popularity, plants with high citation frequencies, availability, and versatility in managing multiple conditions emerged as key species across the surveyed regions. About 42% of the recorded plants (*Boophone disticha*, *Boscia albitrunca*, *Croton gratissimus*, *Entada elephantina*, *Gomphocarpus fruticosus*, *Grewia flava*, *Grewia flavescens*, *Hypoxis hemerocallidea* and *Vachellia karroo*) were identified as the most popularly used plants based on their high citation number (3‐5), availability and/or uses (2‐9) in the management of multiple livestock conditions. The high frequency of citation for most used plants could indicate their effectiveness in managing diverse livestock diseases/conditions, considering that these practices in indigenous knowledge have often been refined over time.

**TABLE 2:** An inventory of ethnobotanical plants used in livestock management by Batswana across southern Africa. The botanical name and families were verified using the Plants of the World Online | Kew Science” (https://powo.science.kew.org/).

Plant species	Family	Local name	Plant part(s) used	Preparation method	Administration mode	Conditions	References
*Acokanthera oppositifolia* (Lam.) Codd	Apocynaceae	Serekolo	Leaves	Decoction	Oral	Internal parasites	[53]
*Acrotome inflata* Benth.	Lamiaceae	Mogato	Leaves, Whole plant	Decoction, Burn	Oral, Topical	Cough, wounds	[76]
			Roots	Infusion	Oral	Wounds, abscess in livestock	[64]
*Aloe ferox* Mill.	Asphodelaceae	Mokgwapha/ Sekgophana	Leaves	Chopped	Oral	NCD, coccidiosis and respiratory diseases	[131]
				Infusion		Internal parasites	[53]
						Snakebite control	[55]
				Unspecified	Unspecified	Diarrhoea, Cough	[63]
		Unspecified				Worms, Diarrhoea, Constipation	[109]
*Aloe greatheadii* Schönland	Asphodelaceae	Kgopane e nyane	Leaves	Decoction, Infusion	Oral	Constipation, diarrhoea, retained placenta, ticks, abscesses, wounds, muscle pain	[76]
				Decoction		Retained placenta, enhance blood circulation and treating diarrhoea	[52]
		Kgophane	Whole plant	Unspecified	Unspecified	Burns, general ailments, blood cleansing, internal parasites, eye infections	[91]
*Aloe marlothii* A. Berger	Asphodelaceae	Unspecified	Unspecified	Unspecified	Unspecified	Unspecified	[171]
		Mokgopa	Leaves			Gallsickness, internal and external parasites, diarrhoea, constipation, retained placenta, dystocia, maggots	[91]
*Aloe vera* (L.) Burm.f.	Asphodelaceae	Kgopane ya thaba	Leaves	Ground, decoction, infusion	Topical, oral	Abscess, wounds, retained placenta, diarrhoea	[76]
				Decoction	Oral	Retained placenta, diarrhoea and gala	[52]
*Aloe zebrina* Baker	Asphodelaceae	Kgophane	Leaves	Infusion, roosted,	Oral, topical	Ripening of abscess, fleas, Gastrointestinal parasites, gala	[64]
			Whole plant	Unspecified	Unspecified	Burns, general ailments, blood cleansing, internal parasites, eye infections	[91]
*Amaranthus blitum* L.	Amaranthaceae	Modinakana	Leaves	Infusion	Oral	Blood cleansing, wounds	[64]
*Amaranthus cruentus* L.	Amaranthaceae	Setlepetlepe	Roots, leaves, whole plant	Poultice, ground	Oral, topical	Abscess, wounds, ear pain	[76]
		Modinakana	Whole plant	Ground	Oral	Constipation	[76]
*Ansellia africana* Lindl.	Orchidaceae	Palamela	Unspecified	Unspecified	Unspecified	Diarrhoea	[56]
			Roots	Decoction	Oral	Internal parasites	[53]
*Aptosimum elongatum* (Hiern) Engl.	Scrophulariaceae	Ditantanyane	Whole plant	Decoction	Oral	Arthralgia	[76]
*Artemisia afra *Jacq. ex Willd.	Asteraceae	Lengana	Leaves	Decoction, ground	Oral, topical	Cough, intestinal worms, arthralgia, ear pain	[76]
				Infusion	Oral	Cough	[64]
*Asparagus africanus* Lam.	Asparagaceae	Thokabotswaro	Roots, Stems	Infusion	Oral	Malnutrition	[76]
*Asparagus laricinus* Burch.	Asparagaceae	Lesitwane	Whole plant	Decoction	Oral	Muscle pain	[76]
			Tubers	Unspecified	Unspecified	Sores, redwater, uterine infection, general ailments, umbilical cord inflammation	[91]
		Mositwasitwane	Roots/nods			Retained placenta	[62]
*Asparagus nodulosus* (Oberm.) J.‐P. Lebrun & Stork	Asparagaceae	Radipolwane/ polopolwane	Root	Decoction	Oral	Eye infection, retained placenta	[64]
*Asparagus suaveolens* Burch.	Asparagaceae	Motantanyane	Whole plant	Decoction	Oral	Dystocia	[76]
		Lesitwane	Tubers	Unspecified	Unspecified	Sores, redwater, uterine infection, general ailments, umbilical cord inflammation	[91]
*Babiana hypogaea* Burch.	Iridaceae	Thuge	Leaves	Infusion	Oral	Abscess, muscle pain	[76]
			Tuber			Diarrhoea	[64]
*Boerhavia diffusa* L.	Nyctaginaceae	Moetapele	Leaves, Stems	Decoction	Topical	Eye infection, abscess, wounds	[76]
*Boophone disticha* Herb.	Amaryllidaceae	Leswama	Bulb	Decoction	Oral Oral	Fracture, post‐abortion, retained placenta	[64]
		Lesoma/ Mathubadudifala	Leaves, Roots, Bulb			Constipation	[76]
		Lesoma (Legwama)	Roots, Leaves	Maceration		Retained placenta and wound healing	[52]
		Matubadifala	Bulb scales	Unspecified	Unspecified	Abortion	[91]
			Unspecified			Retained placenta, gall sickness	[109]
*Boscia albitrunca* (Burch.) Gilg & Benedict	Capparaceae	Motlopi	Leaves, Roots	Decoction, ground	Oral	Internal parasites	[53]
						Unspecified	[131]
				Unspecified	Unspecified	Retained placenta	[62]
			Bark			Anthrax	[56]
						Eye diseases	[63]
*Boscia foetida* Schinz	Capparaceae	Mopipi	Leaves	Ground	Unspecified	Eye problems	[56]
*Bulbine abyssinica* A. Rich.	Asphodelaceae	Makgabenyane	Leaves	Ground	Topical	Abscess, wounds	[76]
			Unspecified	Unspecified	Unspecified	Gall sickness, worms	[109]
			Roots	Infusion	Oral	Blood cleansing, internal sores	[64]
*Burkea africana* Hook.	Fabaceae	Monato	Bark	Unspecified	Unspecified	Retained placenta	[62]
							[63]
*Cadaba aphylla* (Thunb.) Wild	Capparaceae	Monnamontsho	Roots	Decoction	Oral	Blood cleansing	[64]
*Cannabis sativa* L.	Cannabaceae	Motekwane	Leaves	Decoction	Oral	Anthelmintic	[64]
*Capsicum annuum* L.	Solanaceae	Pherehere	Leaves/fruit	Chopped	Oral	Unspecified	[131]
*Cassia abbreviata* Oliv.	Fabaceae	Unspecified	Unspecified	Unspecified	Unspecified	Unspecified	[171]
*Centella asiatica* (L.) Urb.	Apiaceae	Setimamolelo Setimamolelo	Leaves, whole plant	Poultice, decoction	Topical, oral	Wound, abscess, eye infection, diarrhoea	[76]
			Whole plant	Maceration	Oral	Retained placenta	[52]
*Cleome gynandra* L.	Cleomaceae	Rothwe	Flower, leaves, roots	Ground	Topical	Eye infection, ear problem, cough, constipation, intestinal worms	[76]
*Colophospermum mopane* (J. Kirk ex Benth.) J. Léonard	Fabaceae	Mophane	Bark, leaves	Infusion, decoction	Oral	Internal parasites	[53]
*Combretum hereroense* Schinz	Combretaceae	Tsholakhudu	Leaves	Decoction	Oral	Cough, pains, dysentery, constipation	[76]
*Combretum imberbe* Wawra	Combretaceae	Unspecified	Unspecified	Unspecified	Unspecified	Fleas, mites, ticks	[171]
*Croton gratissimus* Burch	Euphorbiaceae	Moologa	Flower	Ground	Topical	Eye infection, ear problem	[76]
			Leaves			Fertility enhancement	[64]
			Leaves, Roots	Unspecified	Unspecified	Pneumonia, fertility enhancement	[91]
*Croton megalobotrys* Müll.Arg.	Euphorbiaceae	Unspecified	Leaves	Unspecified	Topical	Lumpy skin	[56]
*Cucumis myriocarpus* Naudin	Cucurbitaceae	Monyaku	Fruit	Infusion	Oral	Vomiting, general malaise (gala)	[64]
*Dichrostachys cinerea* (L.) Wight & Arn.	Fabaceae	Moselesele	Bark	Poultice	Topical	Retained placenta, dystocia, fracture, arthralgia	[76]
			Roots		Topical	Retained placenta, dystocia, fracture	[52]
*Dicoma galpinii* F.C. Wilson	Asteraceae	Tlhlonya	Roots	Infusion	Oral	Diarrhoea, blood cleansing	[64]
*Dicoma macrocephala* DC.	Asteraceae	Tlhonya	Roots	Infusion	Oral	Diarrhoea	[76]
*Diospyros lycioides* Desf.	Ebenaceae	Motlhajwa/letlhajwa	Roots	Decoction	Oral	Snakebite control	[55]
*Dracaena hyacinthoides* (L.) Mabb.	Asparagaceae	Moshokelatsebe	Leaves, whole plant	Poultice, decoction	Topical, oral	Retained placenta, diarrhoea, constipation	[76]
*Drimia sanguinea* (Schinz) Jessop	Asparagaceae	Sekaname	Bulb	Infusion	Oral	Retained placenta, intestinal worms, constipation	[76]
				Poultice	Oral	General ailments, general intestinal diseases, internal parasites, blood cleansing, gallsickness, heartwater, redwater, sores, retained placenta	[91]
			Roots			Retained placenta, uterus, blood cleaning	[52]
						Snakebite, heartwater	[64]
				Unspecified	Unspecified	Foot rot	[63]
						Gallsickness, worms	[91]
			Unspecified				
*Dysphania ambrosioides* (L.) Mosyakin & Clemants	Amaranthaceae	Tlhatlhabadimo	Whole plant	Infusion	Oral	Cough, constipation	[76]
*Ehretia rigida* Druce	Boraginaceae	Morobe	Roots	Unspecified	Unspecified	Fractures	[91]
*Elaeodendron transvaalense* (Burtt Davy) R.H. Archer	Celastraceae	Mojelemane	Bark	Decoction	Oral	Diarrhoea	[76]
				Unspecified	Unspecified		[91]
*Englerophytum magalismontanum* (Sonder) T.D.Penn.	Sapotaceae	Motlatswa	Roots	Unspecified	Unspecified	Fertility enhancement	[91]
*Entada burkei* (Benth.) S.A. O'Donnell & G.P. Lewis	Fabaceae	Mositsane	Roots, Bark	Decoction, ground	Oral, topical	Cough, constipation, retained placenta, diarrhoea	[76]
*Entada elephantina* (Burch.) S.A. O'Donnell & G.P. Lewis	Fabaceae	Mosetlhane Mositsane Bosetsana	Root‐stock	Unspecified	Unspecified	Diarrhoea, heartwater, coughing, pneumonia	[91]
			Bulb			Retained placenta	[62]
			Roots	Poultice	Topical	Retained placenta, intestinal para sites, enhance blood circulation	[52]
			Leaves	Decoction	Oral	Internal parasites	[53]
			Rhizome	Infusion		Blood cleansing	[64]
*Euclea undulata* Thunb.	Ebenaceae	Morobe	Leaves, bark, roots	Poultice, decoction	Topical, oral	Wounds, cough, constipation, retained placenta diarrhoea, arthralgia	[76]
*Euphorbia balbisii* Boiss.	Euphorbiaceae	Lwetsane	Leaves, Roots	Decoction	Oral	Diarrhoea, intestinal worms	[76]
*Euphorbia inaequilatera* Sond.	Euphorbiaceae	Loetsane	Roots	Infusion	Unspecified	Eye problems	[56]
*Euphorbia regis‐jubae* Webb & Berthel.	Euphorbiaceae	Mosimama/Mosiama	Branches	Ground	Oral, topical	Snakebite control	[55]
*Euphorbia serpens* Kunth	Euphorbiaceae	Luetsane	Roots	Decoction	Oral	Blood cleansing	[64]
*Gomphocarpus fruticosus* (L.) W.T. Aiton	Apocynaceae	Motimola/ sebogamaswi	Whole plant	Infusion	Oral	Constipation, retained placenta, cough, bile reflux	[76]
		Motimola		Maceration		Retained placenta, pain alleviation	[52]
		Sebogamashi	Roots	Decoction		Retained placenta, gala, respiratory diseases	[64]
*Grewia flava* DC.	Malvaceae	Moretlwa	Roots	Infusion	Oral	Diarrhoea, dystocia	[76]
				Decoction		Diarrhoea	[64]
				Unspecified	Unspecified	Fertility enhancement	[91]
*Grewia flavescens* Juss.	Malvaceae	Mokgompata	Unspecified	Unspecified	Unspecified	Diarrhoea	[56]
		Mokgomphatha	Roots			Foot rot	[63]
		Motsotsojane	Leaves	Infusion	Oral	Pain, wounds, diarrhoea	[76]
			Leaves, Roots			Internal parasites	[53]
*Harpagophytum procumbens* (Burch.) DC. ex Meisn.	Pedaliaceae	Sengaparile	Roots	Unspecified	Unspecified	Mange	[63]
		Lematla, Sengaparile	Fruit	Decoction, ground	Oral, topical	Retained placenta	[91]
			Tuber, roots, leaves, fruit			Dystocia, pain after birth, abscess, fracture, muscle pain, retained placenta	[76]
*Helichrysum candolleanum* H.Buek	Asteraceae	Phateyangaka Phate ya ngaka	Roots, leaves, fruit	Decoction	Oral	Retained placenta	[76]
			Unspecified	Unspecified	Unspecified	Fowl pox, swelling of the head	[109]
*Helichrysum paronychioides* DC.	Asteraceae	Phateyangaka	Roots	Infusion	Oral	Cough, blood cleansing, pain, diarrhoea	[64]
*Hermannia guerkeana* K. Schum.	Malvaceae	Moreba	Roots	Unspecified	Unspecified	Retained placenta	[62]
*Hypoxis hemerocallidea* Fisch., C.A. Mey. & Avé‐Lall.	Hypoxidaceae	Maledu/Tshuku ya poo	Whole plant	Decoction	Oral	Cough, dystocia, arthralgia, constipation	[76]
			Unspecified	Unspecified	Unspecified	Gall sickness	[109]
			Corms			Fertility enhancement, general ailments, heartwater, abortion	[91]
			Bulb	Poultice	Topical	Retained placenta, anaemia	[52]
*Hypoxis rigidula* Baker	Hypoxidaceae	Tsuku‐ya‐poo	Corms	Unspecified	Unspecified	Fertility enhancement, general ailments, heartwater, abortion	[91]
*Indigofera cryptantha* Benth. ex Harv.	Fabaceae	Kofi	Roots	Decoction	Oral	Diarrhoea	[64]
*Ipomoea oblongata* E. Mey. ex‐Choisy	Convolvulaceae	Mokatelo	Roots	Decoction	Oral	Cough, wounds, muscle pain, diarrhoea	[76]
*Jatropha zeyheri* Sond.	Euphorbiaceae	Seswagadi	Roots	Maceration	Topical	Eye infections, constipation, retained placenta	[76]
				Poultice		Retained placenta, blood cleansing and kidney stone	[52]
*Kleinia longiflora* DC.	Asteraceae	Mosimama Mosiama	Whole plant	Poultice	Topical	Eye infection	[76]
				Ground		Fracture	[64]
*Lasiosiphon capitatus* (Lam.) Burtt Davy	Thymelaeaceae	Mokaikai	Unspecified	Unspecified	Unspecified	Diarrhoea	[56]
			Roots, leaves	Decoction, infusion	Oral	Internal parasites	[53]
*Lippia scaberrima* Sond.	Verbenaceae	Mosukutswane	Leaves	Decoction	Oral	Cough	[76]
*Lycianthes biflora* (Lour.) Bitter	Solanaceae	Makgonatsotlhe	Roots	Infusion	Oral, topical	Intestinal worms	[76]
					Oral	Internal parasites	[53]
*Malva neglecta* Wallr.	Malvaceae	Tikamotse	Leaves, flowers	Decoction	Oral	Constipation, wounds, abscess, cough	[76]
*Malvastrum coromandelianu*m (L.) Garcke	Malvaceae	Thobega	Leaves	Decoction	Oral	Diarrhoea, abscess, wounds, ear pain	[76]
*Mentha aquatica* L.	Lamiaceae	Kgobedimetsing	Leaves	Decoction	Oral	Cough	[76]
*Moringa oleifera* Lam.	Moringaceae	Unspecified	Leaves	Ground	Oral	Unspecified	[131]
			Whole plant	Unspecified	Unspecified	Cough	[63]
*Nicotiana tabacum* L.	Solanaceae	Motsoko	Leaves	Grounded	Oral	NCD, coccidiosis and respiratory diseases	[131]
				Unspecified	Unspecified	Eye infections	[91]
		Tobacco				Worms, foaming from the mouth	[109]
						Internal parasites, eye diseases	[63]
*Opuntia ficus‐indica *(L.) Mill.	Cactaceae	Toorofeye	Leaves, stem, flowers	Decoction, Ground	Oral, topical	Diarrhoea, constipation, eye infections, retained placenta, abscess	[76]
			Flower	Poultice	Topical	Retained placenta	[52]
*Osyris lanceolata* Hochst. & Steud.	Santalaceae	Mpera	Bulb	Maceration	Oral	Retained placenta, alleviation of pain, internal bleeding	[52]
*Ozoroa paniculosa* (Sond.) R. Fern. & A.Fern.	Anacardiaceae	Monokana Monokane	Roots	Decoction	Oral	Cough, muscle pain	[76]
				Unspecified	Unspecified	Retained placenta	[62]
			Bark, rootbark			Diarrhoea, redwater, sweating sickness	[91]
*Peltophorum africanum* Sond.	Fabaceae	Mosetlha Mosetla Unspecified	Roots, bark	Decoction	Oral	Wounds, muscle pain, diarrhoea, constipation	[76]
			Roots, leaves, bark			Internal parasites	[53]
			Leaves, Bark	Poultice	Topical	Retained placenta diarrhoea and removal of blood clots from the skin	[52]
			Roots	Unspecified	Unspecified	Retained placenta	[62]
			Bark, rootbark			Diarrhoea	[91]
			Unspecified	Unspecified		Fleas, mites, ticks	[171]
*Phyllanthus maderaspatensis* L.	Phyllanthaceae	Mositwane	Whole plant	Ground, Decoction	Topical, Oral	Eye infection, constipation, diarrhoea	[76]
*Phyllanthus parvulus* var. garipensis (Müll.Arg.) Radcl.‐Sm.	Phyllanthaceae	Lentsane	Aerial parts	Unspecified	Unspecified	Eye infections	[91]
*Phyllanthus parvulus* Sond.	Phyllanthaceae	Lentsane	Aerial parts	Unspecified	Unspecified	Eye infections	[91]
*Plumbago zeylanica* L.	Plumbaginaceae	Masegomabe	Whole plant	Decoction	Oral	Cough, intestinal worms	[76]
			Roots	Unspecified	Unspecified	Pneumonia	[91]
*Portulaca oleracea* L.	Portulacaceae	Selele	Whole plant	Decoction	Oral	Constipation, eye infection, muscle pain, wounds, intestinal worms	[76]
*Pouzolzia mixta* Solms	Urticaceae	Mongololo	Roots, leaves	Maceration, decoction, infusion	Oral	Retained placenta, diarrhoea, constipation	[76]
				Unspecified	Unspecified	Retained placenta, bloat, vaginal discharge	[91]
			Roots	Poultice	Topical	Retained placenta, uterus cleansing	[52]
				Unspecified	Unspecified	Retained placenta	[62]
*Rhoicissus tridentata* (L.f.) Wild & R.B. Drumm.	Vitaceae	Ntagaraga	Tubers	Unspecified	Unspecified	Heartwater, redwater, internal parasites, general ailments, abortion	[91]
*Ricinus communis* L.	Euphorbiaceae	Mokhura	Leaves	Infusion	Oral	Constipation, eye infection	[76]
			Seeds	Unspecified	Unspecified	Constipation, internal parasites	[91]
*Scadoxus puniceus* (L.) Friis & Nordal	Amaryllidaceae	Sekaname	Roots	Unspecified	Unspecified	Retained placenta	[62]
*Schkuhria pinnata *(Lam.) Kuntze ex Thell.	Asteraceae	Santlhoko Santhloko, Lefero	Whole plant	Ground	Topical	Eye infection, wounds, abscess	[76]
			Aerial parts	Unspecified	Unspecified	Eye infections, pneumonia, diarrhoea, heartwater	[91]
*Sclerocarya birrea* Hochst.	Anacardiaceae	Morula	Barks	Unspecified	Unspecified	Diarrhoea, fracture	[91]
*Searsia lancea* (L.f.) F.A. Barkley	Anacardiaceae	Moshabela Moshabele	Roots, leaves, stem	Poultice, infusion	Oral	Abscess, constipation, diarrhoea	[76]
			Roots, bark	Unspecified	Unspecified	Diarrhoea, gallsickness	[91]
*Searsia pyroides* (Burch.) Moffett	Anacardiaceae	Bohitlha	Leaves	Decoction	Oral	Cough, dystocia, constipation, diarrhoea. intestinal worms, arthralgia	[76]
			Roots	Poultice	Topical	Retained placenta	[52]
*Securidaca longepedunculata* Fresen.	Polygalaceae	Mmaba	Roots	Ground	Topical	Cough, dystocia, constipation, muscle pain	[76]
*Seddera suffruticosa* Hallier f.	Convolvulaceae	Thobega	Roots	Unspecified	Unspecified	Fracture	[91]
*Senecio consanguineus* DC.	Asteraceae	Unspecified	Whole plant	Decoction	Oral	Cough, wounds, constipation	[76]
*Senna italica* Mill.	Fabaceae	Sebetebete/Sebete/Okatare	Leaves, bark	Decoction	Oral	Constipation, abscess, anthrax, aphosphorosis, lung diseases	[76]
		Unspecified	Roots	Poultice	Topical	Retained placenta, pain alleviation	[52]
				Unspecified	Unspecified	Gallsickness, general intestinal diseases, heartwater, anthrax, pneumonia	[91]
			Whole plant, roots			Diarrhoea, retained placenta	[64]
			Roots, Whole plant			Pasteurollosis, diphtheria	[63]
			Unspecified	Infusion chopped		Liver disease, gallsickness	[109]
						Calf diphtheria	[56]
						Unspecified	[171]
		Monyokololo				Gall sickness, worms	[109]
			Leaves/roots			Unspecified	[131]
*Senna tora* (L.) Roxb.	Fabaceae	Mongepenpe	Whole plant	Poultice	Topical	Retained placenta, growth of scrotum	[52]
*Sesamum eriocarpum* (Decne.) Byng & Christenh.	Pedaliaceae	Makanangwane	Roots	Unspecified	Unspecified	Retained placenta	[62]
		Tshetlho ya mibitlae mebedi	Whole plant	Poultice	Topical		[63]
						Retained placenta, dystocia, general ailments	[91]
						Retained placenta, flea eradication	[52]
		Tshetlho ya mamitlwa a mabedi	Leaves, whole plant roots	Poultice, infusion	Topical, oral	Blackquarter, retained placenta, dystocia	[76]
		Makanangwane		Unspecified	Unspecified	Retained placenta	[62]
*Solanum campylacanthum* Hochst. ex A. Rich.	Solanaceae	Tolwane enyane Tholwane e nyane	Roots, leaves	Infusion, maceration	Oral	Diarrhoea, eye infection	[76]
			Roots	Decoction		Blood cleansing	[64]
*Solanum dimidiatum* Raf.	Solanaceae	Mohato	Fruit sap	Unspecified	Unspecified	Diarrhoea	[91]
*Solanum albidum* Dunal	Solanaceae	Tolwana	Roots	Unspecified	Unspecified	Sores	[91]
*Solanum lichtensteinii* Willd.	Solanaceae	Tolwane	Whole plant	Poultice	Topical	Ticks	[76]
			Flower, roots			Retained placenta	[52]
		Tholwane	Roots	Infusion	Oral	Blood cleansing, gastrointestinal parasites	[64]
*Spirostachys africana* Sond.	Euphorbiaceae	Morukuru	Bark	Unspecified	Unspecified	Retained placenta	[62]
							[63]
		Morekhure	Stem			Sweating sickness	[91]
*Tarchonanthus camphoratus* Houtt. ex DC.	Asteraceae	Moologa	Leaves	Maceration	Oral	Retained placenta, wounds, dystocia	[52]
*Tarchonanthus camphoratus* L.	Asteraceae	Moologa	RootsLeaves	Infusion	Oral	Internal parasites	[53]
		Mohatlha				Intestinal worms	[76]
						Cold	[64]
*Terminalia sericea* Burch. ex DC.	Combretaceae	Mogonono	Leaves, stem	Decoction	Oral	Cough	[76]
			Roots	Poultice	Topical	Retained placenta, uterus cleansing	[52]
				Unspecified	Unspecified	Diarrhoea	[63]
							[91]
							[56]
			Root bark			Retained placenta	[62]
			Leaves				[62]
			Unspecified	Infusion	Oral	Internal parasites	[53]
*Teucrium trifidum* Retz.	Lamiaceae	Lethe la noga	Leaves, roots	Decoction	Oral	Cough, diarrhoea, constipation	[76]
			Whole plant	Unspecified		Maintenance of pregnancy after abortion	[64]
*Thamnosma rhodesica* (Baker f.) Mendonça	Rutaceae	Moralala	Whole plant	Unspecified	Unspecified	Contagious abortion	[63]
*Thesium viridifolium* Levyns	Santalaceae	Motlhogapele	Whole plant	Decoction	Oral	Diarrhoea	[64]
*Tribulus terrestris* L.	Zygophyllaceae	Tshetlho Tsetlho Tshetlo	Leaves, whole plant	Ground	Oral	Arthralgia,	[76]
			Whole plant	Poultice	Topical	Retained placenta, wound healing, dystocia	[52]
				Unspecified	Unspecified	Retained placenta, bloat	[91]
*Triumfetta sonderii* Ficalho & Hiern	Malvaceae	Mokuku	Rootbark	Unspecified	Unspecified	Retained placenta	[91]
*Vachellia karroo* (Hayne) Banfi & Galasso	Fabaceae	Mooka Mookana	Bark	Decoction	Oral	Lumpy skin disease	[76]
			Bulb	Maceration		Retained placenta, bacterial infection	[52]
			Bark	Unspecified	Unspecified	Fractures, diarrhoea	[91]
			Root, bark	Ground	Topical	Fracture	[64]
*Vachellia tortilis* (Forssk.) Galasso & Banfi	Fabaceae	Mosu	Branch tips	Unspecified	Unspecified	Diarrhoea	[91]
*Vitex zeyheri* Sond. ex Schauer	Lamiaceae	Mokwele	Leaves	Unspecified	Unspecified	Eye infections	[91]
*Withania somnifera* (L.) Dunal	Solanaceae	Modikasope Mokukwane	Roots	Infusion	Oral	Internal sores	[64]
				Unspecified	Unspecified	Diarrhoea	[91]
*Ximenia americana* L.	Olacaceae	Moretologana	Unspecified	Unspecified	Unspecified	Diarrhoea	[56]
		Seretologa	Roots			Internal parasites	[91]
*Ziziphus mucronata* Willd.	Rhamnaceae	Mokgalo/Sekgalo	Leaves	Poultice	Topical	Abscess	[64]
			Roots			Retained placenta	[52]
				Decoction	Oral	Snakebite control	[55]
				Unspecified	Unspecified	Retained placenta	[62]
			Unspecified			Diarrhoea	[56]
			Roots, leaves			Fertility enhancement, sores, burns	[91]
				Decoction, ground	Oral, topical	Dystocia, diarrhoea, arthralgia, wounds, foot rot	[76]
				Decoction, infusion	Oral	Internal parasites	[53]
*Ziziphus oxyphylla* Edgew.	Rhamnaceae	Mokgalo fatshe	Roots	Decoction	Unspecified	Diarrhoea	[109]
		Sekgalofatshe		Poultice	Topical	Retained placenta, increase stimulation for separating retained placenta	[52]
*Ziziphus zeyheriana* Sond.	Rhamnaceae	Sekgalofatshe/Mokgalofatshe	Roots	Decoction	Oral	Blood cleansing, pain	[64]
		Sekgalo‐fatshe	Leaves, branches	Unspecified	Unspecified	Diarrhoea, internal parasites, general ailments	[91]

#### 3.2.2 Distribution of Plant Families Used to Manage Livestock Health Conditions

**FIGURE 2:**
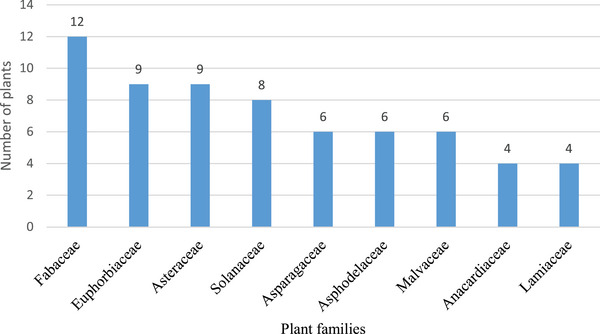
Distribution of plant families (with ≥ 4 mentioned plants) used to manage livestock health conditions among the Batswana in southern Africa. In addition to the top nine mentioned family, we recorded 35 families with plants ranging from 1–3 (See Table 2).

The recorded 116 plants were distributed within 44 families with the Fabaceae (12), Euphorbiaceae (9), Asteraceae (9), Solanaceae (8), Asparagaceae (6), Asphodelaceae (6) and Malvaceae (6) having the highest cited number of plants used to manage livestock conditions among Batswana people in southern Africa (Figure 2 and Table 2). Similarly, the high utilisation of the Fabaceae in managing different livestock conditions has been reported in ethnobotanical reviews or studies conducted in Africa [44, 45, 46]. The top 10 families comprised 56.89% of the total cited plants, while the remaining (42.24%) plants were represented within 34 other families. Furthermore, 84.09% of the families had relatively low representation averaging 1–4 plant species per family. The prevalent use of the Fabaceae family may likely be attributed to its broad distribution, high species richness, and diverse bioactive compounds known for their pharmacological properties [47, 48, 49]. This diversity reflects the broad spectrum of traditional plant knowledge across southern Africa, where various families are utilised for their specific benefits in livestock health management.

#### 3.2.3 Pattern of Plant Parts Used, Preparation, and Route of Administration Methods

**FIGURE 3:**
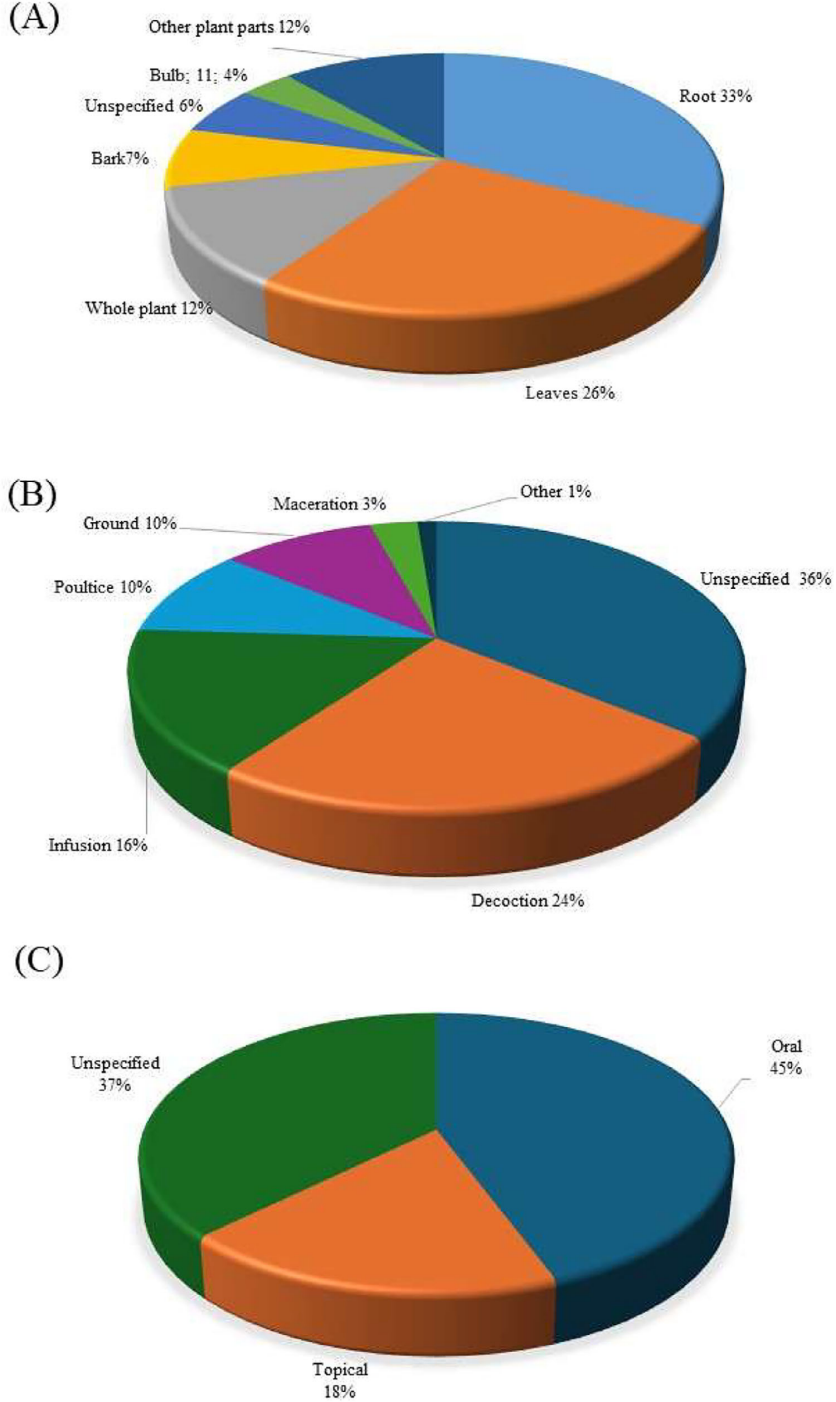
Distribution of different parameters associated with plants utilised for managing livestock health conditions among the Batswana in southern Africa. A—plant parts (*n* = 298); B‐ preparation methods (*n* = 264); and C‐ administration mode (*n* = 257).

A total of 15 plant parts were used for treating livestock diseases among Batswana in southern Africa (Table 2). The most common plant parts used were roots (33%), leaves (26%), and whole plant (12%) (Figure 3A, Table S1). The popularity of roots as one of the most preferred plant parts has led to significant conservation challenges. The harvest of underground parts as a practice is often unsustainable, causing irreversible damage to plant populations and contributing to the risk of species decline which can lead to extinction [50, 51]. The dominance of root usage in Batswana ethnoveterinary practices may be attributed to their belief in the strength and vitality that the earth imparts to these underground parts [52, 53, 54]. Roots and leaves are the most frequently used, reflecting traditional preferences for these accessible and widely applicable plant parts [13, 55, 56].

The methods of preparing medicinal plants for livestock conditions in southern Africa highlight a range of traditional techniques tailored to different health conditions [57]. Decoctions (24%) and infusions (16%) were the most common preparation methods used for medicinal plants among Batswana in southern Africa (Figure 3B). Decoction entails boiling the plant materials while infusion involves pouring cold/hot/warm water onto the plant material and allowing the mixture to steep and cool. Furthermore, poultices (10%) are primarily used for external treatments including wound care, skin infections and inflammation. This method involves crushing plant materials and applying them directly to the affected area. Grinding of the plant materials constituted 10% of the reported preparation methods. On the other hand, methods of preparation such as maceration (3.4%), burning (0.38%) and roasting (0.38%) were generally low (Table S2). It is important to highlight that a significant portion (36%) of the plant preparations do not have the specific method used. This could be due to traditional practices where the method is considered implicit or universally understood within the communities.

In southern African ethnoveterinary practices, the mode of administering medicinal plants varies significantly, with oral administration (44%) and topical applications (19%) being the most frequently cited practices (Figure 3C). Oral administration is favoured for treatments targeting internal ailments (e.g., organ damage, inflammation, infections), and topical applications is a localised practice used for conditions such as wounds and skin infections, offering a targeted approach for external relief [58]. The high percentage of unspecified administration (37%) suggests some flexibility in traditional practices, where the method may depend on the practitioner's preference or the circumstances of each treatment. This distribution of administration routes highlights the adaptability and specificity of traditional livestock health condition treatment approaches [59].

#### 3.2.4 Livestock Health Conditions Treated With Plants by the Batswana in Southern Africa

A total of 58 livestock conditions identified were categorised into 10 major groups (Table 3). The classification of the different diseases was based on the studies by Chakale et al. [60] and Ndou [61], with some slight modifications. Some of the dominant categories included, reproduction disorders (121), gastrointestinal problems (97), and skin problems (74). On the other hand, treatment of conditions such as eye problems and musculoskeletal systems were relatively lower in significance. This may reflect a lower prevalence of these issues or the possibility that such conditions are managed through other methods or external interventions beyond traditional plant‐based remedies. Retained placenta (81), diarrhoea (65), and wounds (44) were the most cited conditions managed within the livestock conditions (Table 3). Among these, retained placenta emerged as the most cited health condition. This underscores the importance of managing reproductive health in livestock, as issues such as retained placenta can significantly affect the productivity and reproductive efficiency of animals [15, 52, 62]. The frequent citation of gastrointestinal disorders shows the critical need for remedies to ensure digestive health, as poor digestion can lead to reduced nutrient absorption, weight loss, and lower productivity in livestock [56, 63]. Skin problems were also prominent, with eight conditions cited, including wounds. The high number of references to wound treatment suggests that topical application of plant‐based remedies is a crucial aspect of traditional veterinary care [14, 64]. The wound‐related problems may reflect the challenges posed by injuries sustained during grazing, handling, or attacks by predators, making wound care an essential aspect of livestock management [13].

**TABLE 3:** Livestock health conditions managed with medicinal plants by the Batswana in southern Africa.

Category of the conditions	Conditions	Number of plants used
Eye problem	Eye infection	19
	Conjunctivitis	3
	Blindness	1
Fertility/reproduction disorders	Retained placenta	81
	Dystocia	11
	Abortion	10
	Fertility problems	4
	Uterus cleansing	3
	Bloat	2
	Pain after birth	1
	Growth of scrotum	1
	Urinary infections	1
	Vaginal discharge	1
Gastrointestinal problems	Diarrhoea	65
	Constipation	29
	Bile reflux	6
	Gastrointestinal parasites	2
	Dysentery	1
	Kidney stone	1
General system infection	Blood cleansing	13
	Anthrax	6
	Black quarter	6
	Ear pain	5
	Enhance blood circulation	2
	Aphosphorosis	1
	Malnutrition	1
	Weating sickness	1
Internal and external parasites	Internal parasites	19
	Helminths	13
	Babesiosis	6
	Intestinal worms	6
	Anaemia	3
	Coccidiosis	2
	Newcastle Disease (NCD)	2
	Bacterial infection	1
Musculoskeletal systems	Fracture	13
	Pain	12
	Arthralgia	7
Respiratory problems	Cough	26
	Pneumonia	3
	Respiratory diseases	3
	Diphtheria	2
	Heart problem	1
	Lung diseases	1
Skin problem	Wounds	44
	Abscess	16
	Sores	5
	Foot rot	4
	Lumpy skin	2
	Pasteurollosis	1
	Mange	1
	Myiasis	1
Snakebite	Snakebite control	5
Tick‐borne	Anaplasmosis	23
	Cowdriosis	8
	Ticks	6
	Fleas	4
	Mites	2
	Heartwater	2
Unspecified	Unspecified	10

The widespread use of medicinal plants reflects both their accessibility and cultural significance, demonstrating how southern African communities have developed adaptive strategies for livestock disease management. Research underscores the potential of medicinal plants in ethnoveterinary practices, emphasising the need for systematic evaluations of their biological and pharmacological effects [65, 66]. Further investigation into the efficacy and safety of these plant‐based treatments could enhance their application and facilitate their integration into sustainable livestock management practices. Additionally, systematic documentation and conservation efforts are essential to ensure the continued availability of these medicinal plant species for future generations [67].

#### 3.2.5 Cultural Significance of Local Names for Plants Among the Batswana of Southern Africa

Among the Batswana communities in South Africa and Botswana, plants play a crucial role in ethnoveterinary medicine, with local names serving as key identifiers in traditional healing practices. These indigenous names encapsulate generations of botanical knowledge, reflecting the deep relationship between the people and their environment [68]. Local nomenclature provides valuable insights into plant characteristics, including their medicinal applications, ecological adaptations and distinctive morphological and sensory features such as size, shape, taste, smell and habitat [69].

The naming of medicinal plants among the Batswana is rooted in observation and cultural significance, with each name often describing a particular attribute or use of the plant. For instance, *Senna italica* (Sebete/Sebetebete) is named based on its use as a purgative to treat digestive disorders in livestock, while *Hypoxis hemerocallidea* (Tshuku ya poo) is recognised for its immune‐boosting properties and treatment of infections in cattle. Similarly, *Grewia flava *(Moretlwa) is applied to wounds due to its antibacterial effects, and *Aloe ferox* (Mokgwapha) is valued for alleviating respiratory infections in goats and cattle (Table 2).

The classification of medicinal plants among the Batswana communities in South Africa and Botswana is often inconsistent. In some instances, a single plant species may be identified by multiple local names within the same region. For example, *Entada elephantina* is known by three different names in various areas of South Africa and Botswana which are Mosetlhane, Mositsane and Bosetsana. Conversely, a single local name can be used to describe multiple plant species, leading to potential confusion in plant identification and application. For instance, the name *Sekgalofatshe* is associated with different species, including *Ziziphus oxyphylla* and *Ziziphus zeyheriana*.

### 3.3 Biological Activity, Safety Status and Phytochemicals of Medicinal Plants With Ethnoveterinary Records Among the Batswana of Southern Africa

After establishing the inventory of 116 plants used by the Batswana to manage their livestock, existing evidence on the biological effects, safety assessments and the phytochemicals of these botanicals were assessed. This was essential to identify plants with empirical data and potential for further research especially on their valorisation.

#### 3.3.1 Biological Activity of Medicinal Plants With Ethnoveterinary Records

**FIGURE 4:**
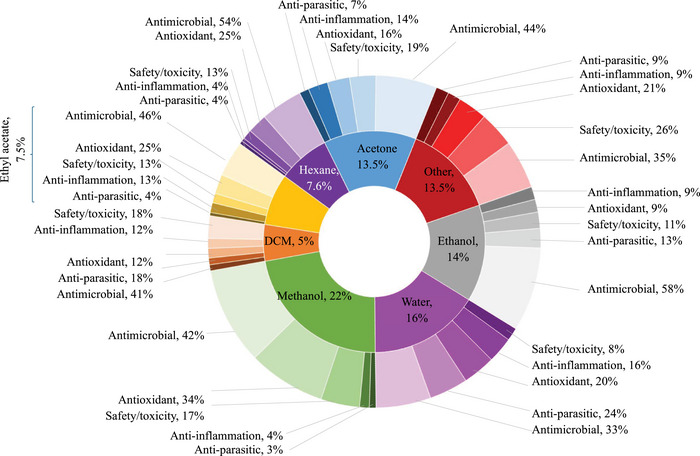
Overview of the solvent extracts used for biological analysis of plants with ethnoveterinary records among the Batswana in southern Africa. Other solvents used that were reported in lower frequencies include petroleum ether, chloroform, and DCM (dichloromethane).

**FIGURE 5:**
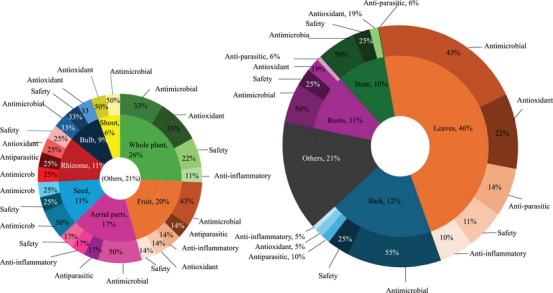
The percentage of plant parts used for the analysis of the biological activities of plants with ethnoveterinary records among the Batswana in southern Africa.

**FIGURE 6:**
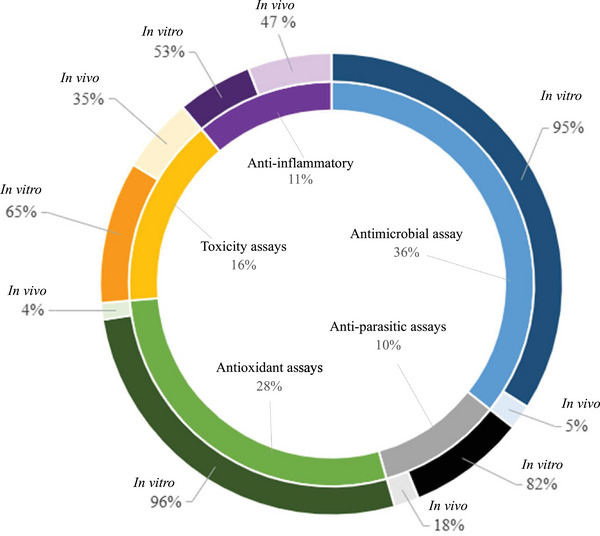
The percentage of the types of bioassays reported for plants with ethnoveterinary records among the Batswana in southern Africa.

Herbal remedies are the oldest form of medication, generally used as multi‐target agents. As of 2024, approximately 3 780 plants have been recorded for medicinal purposes in South Africa [70]. However, there are no recent updates on the number of plants used in ethnoveterinary medicine [71]. Over 60 plants used by the Batswana people have been previously analysed for biological properties related to ethnoveterinary and their phytochemical composition (Tables 4, 5, 6, 7, 8, 9, Figures 4, 5, 6). Traditional medicine covers interdisciplinary research which involves observation, description and conducting experimental analysis of the identified medicinal plants for drug discovery. During the observation stages, plant part usage is crucial as medicinal plants have different kinds of bioactive compounds that accumulate in specific organs at different concentrations. As depicted in Figure 5, leaves were the most studied plant parts (46%) due to their availability, accessibility and plant conservation concerns, even though their frequency of use was 26% in the ethnoveterinary surveys reported (Figure 3A). Availability of plant materials and the complexities of bioactive compounds contribute to the high use of leaves [72]. In the documented ethnoveterinary surveys, roots were the most frequently used (33%) plant part by the Batswana communities, yet leaves accounted for 11% of the plant parts studied (lower than bark usage, 12%) in biological, safety and phytochemical analysis. The low use of seeds and fruits may be attributed to their seasonal availability [73]. Similar findings of limited usage of fruits and seeds were observed in the literature surveys.

**TABLE 4:** Summary of reported antimicrobial activity of plants used by Batswana for livestock health management.

Plant species	Plant part(s) Used	Extraction solvent	Bioassay	Strains tested	Summary of findings	References
*Acrotome inflata* Benth	Fruit	Ethanol	Microplate serial dilution Agar disk diffusion	*Candida albicans *	Fruit extracts were effective against *Candida* species with MIC = 1.25 mg/mL. In the disk diffusion assay, extracts had an inhibition zone of ±2 mm against *C. albicans*. Control: Fungazole and Ampicillin	[90]
*Acrotome inflata* Benth	Whole plant	Ethanol, water	Agar disk diffusion	*Staphylococcus aureus, Pseudomonas aeruginosa*	Ethanolic extracts (whole plant) with concentrations of 5 mg/mL were effective against *P. aeruginosa* (8.7 mm inhibition zone) strain, with 10 mg/mL and 20 mg/mL demonstrating an inhibition zone of ± 11 mm against *B. subtilis*. Control: Streptomycin and Penicillin G	[172]
*Aloe ferox* Mill*. *	Leaves	Methanol, water	Broth microdilution assay	*Staphylococcus aureus, Escherichia coli, Enterococcus faecalis, Bacillus cereus, Bacillus pumilus, Pseudomonas aeruginosa, Klebsiella pneumoniae, Enterococcus cloacae*	Methanol extracts were active against *S. aureus* and *E. coli* at 5 mg/mL MIC. Control: Tetracycline	[129]
*Aloe marlothii* A. Berger	Leaves	DCM, methanol, acetone, hexane, ethanol	Microplate serial dilution TLC Bioautography	*Escherichia coli, Enterococcus faecalis, Pseudomonas aeruginosa, Staphylococcus aureus, Mycobacterium aurum*	All extracts had efficacy against the tested pathogens, with hexane extracts having the highest MIC value (2.5 mg/mL) against all the pathogens. Methanol extract also had an MIC value of 2.5 mg/mL against *E. faecalis* strain. Acetone, DCM and methanol extracts had significant activity against the test pathogens, with MIC values ranging from 0.028 mg/mL (acetone extract against *S. aureus*) and 0.625 mg/mL for methanol extracts against *E. coli*. Leaf extracts exhibit anti‐mycobacterial activity, MIC = 2.5 mg/mL Control: Gentamycin	[89, 90]
*Aloe zebrina *	Leaves	DCM, hexane, acetone, methanol	Microplate serial dilution, bioautography	*Escherichia coli, Enterococcus faecalis, Pseudomonas aeruginosa, Staphylococcus aureus*	Hexane extracts showed the highest activity (2.5 mg/mL) against the tested pathogens. Acetone and methanol extracts showed good activity (0.039 mg/mL and 0.625 mg/mL) against *S. aureus* and *E*. *coli*, respectively. Control: Gentamycin	[89]
*Ansellia africana* Lindl.	Roots, stem	DCM, acetone, DCM‐methanol, water	Agar diffusion assay	*Klebsiella pneumonia, Staphylococcus aureus, Mycobacterium smegmatis, Pseudomonas aeruginosa*	Acetone root extracts inhibited growth of all studied strains, with the highest inhibition zone demonstrated against *S. aureus* (19.3 mm). Root and stem water extracts were not active against the test strains. Root and stem extracts from DCM were as only effective against *K. pneumonia* and *S. aureus*. Control: Ciprofloxacin	[173, 174]
*Artemisia afra* Jacq. ex Willd.	Aerial parts	Ethanol extract	*In vitro* Microplate serial dilution *In vivo* Oral administration of extract	*Salmonella enterica* subsp. *enterica serovar Typhi, Salmonella enterica* subsp. *enterica serovar Enteritidis*	*In vitro* Extracts inhibited *S. enterica* strains and the activity was recorded at 156 µg/mL, and more than 50% biofilm reduction for all the strains. *In vivo* Extracts had a significant reduction in bacterial load of rats show when tested at 200 and 300 mg/kg/bw.	[95]
*Artemisia afra J*acq. ex Willd.	Leaves	Water, DCM	*In vitro* Firefly bioluminescence assay (via optical densitometry at 600 nm) *In vivo* Oral administration of extract	*Mycobacterium tuberculosis H34Rv* *Mycobacterium aurum*	*In vitro* The inhibitory activity of the DCM extract exhibited an IC_50_ = 270 mg/mL when tested against *Mycobacterium aurum* and an IC_50_ = 290 mg/mL for *M. tuberculosis*. The *Mycobacterium aurum* replication was inhibited by 200 µg/mL of water (>25%), methanol (<25%), and DCM extracts (41,4%). Control: Isoniazid (20 µg/mL). *In vivo* No observed improvement of pulmonary burden and spleen burden, indicating no *in vivo* mycobacterial activity	[96]
*Asparagus laricinus* Burch*. *	Stem, leaves	Water	Agar dilution method	*Staphylococcus aureus, Staphylococcus saprophyticus, Enterobacter cloacae*	Leaf extracts exhibited antibacterial activity against *S. aureus* and *B. subtilis* (MIC = 1 mg/mL) as well as *S. saprophyticus* and *E. cloacae* (MIC = 0.125 mg/mL). Control: Chloramphenicol	[175]
*Bulbine abyssinica* A. Rich*. *	Leaves, rhizome, roots	Methanol	Agar well diffusion	*Staphylococcus aureus, Escherichia coli*	The leaf extracts and stems had the greater inhibition against *S. aureus* (inhibition zones of 19.33 and 15 mm respectively), than *E. coli* (inhibition zones of 13.67 and 14.67 mm, respectively). The roots had higher inhibition against *E. coli* (13.67 mm) than against *S. aureus* (12.67 mm) Negative control: DMSO	[176]
*Bulbine abyssinica* A. Rich*. *	Whole plant	Acetone, water	Agar well diffusion assay Microplate serial dilution assay	*Pseudomonas aeruginosa, Staphylococcus aureus, Enterococcus faecalis, Klebsiella pneumonia, Serratia marcescens*	The acetone and water extracts inhibited bacterial growth, particularly the inhibition zones for *E. faecalis* (35 and 41 mm respectively) are greater that the inhibition zone for the positive control (30.67 mm). The inhibition zones for extracts against *P. aeruginosa, S. aureus, K. pneumonia*, and *S. marcescens* strains were lower than the inhibition zone for the positive control. Control: Amoxicillin (0.0125 mg/mL)	[39]
*Bulbine latifolia* (L.f.) Spreng.	Leaves	Methanol	Microplate assay Antitubercular rapid radiometric assay	*Citrobacter, Klebsiella pneumonia, Staphylococcus aureus, Candida albicans, Microsporum audouini, Mycobacterium smegmatis*	The 10 mg/mL extract was effective against *Citrobacter* (MIC = 625 µg/mL), *C. albicans* (MIC = 625 µg/mL), and *M. audouinii* (MIC = 312.5 µg/mL). Controls: Nyastatin (fungi), Gentamycin (bacteria), and Ciprofloxacin & isoniazid (*M. smegmatis*)	[177]
*Cannabis sativa* L.	Leaves	Hexane, DCM, ethyl acetate, ethanol, water	Agar well diffusion assay	*Bacillus cereus, Salmonella enterica*	The extracts were effective against *B. cereus* stain (MIC = 2 mg/mL).	[178]
*Cassia abbreviata* Oliv*. *	Stem Bark	Ethanol, water	Agar disc diffusion	*Escherichia coli*, *Staphylococcus aureus*	Ethanol extracts showed no inhibitory activity against *E. coli* at all tested concentrations (1, 5, 10, 15, 20 mg/mL). Control: Ciprofloxacin (5 µg)	[179]
*Cassia abbreviata* Oliv.	Stem bark	Ethanol, water Soxhlet (cold ethanol, cold water, DCM, Trichloromethane (TCM): ethanol)	Agar well diffusion assay	*Pseudomonas aeruginosa, Klebsiella pneumonia, Candida albicans*	Water extract was active against *P. aeruginosa* (46.88 µg/mL). The TCM extract was active against *K. pneumonia* with an MIC = 46.88 µg/mL. The ethanol extracts showed activity against *C. albicans* with an MIC = 93.75 µg/mL. Controls: Ceftriaxone, Ciprofloxacin, Fluconazole	[92]
*Cassia abbreviata* Oliv*. *	Leaf, stem bark, root bark	Ethanol	Microplate serial dilution assay	*Escherichia coli, Salmonella paratyphi, Klebsiella pneumoniae, Shigella sonnei, Enterobacter cloacae, Pseudomonas aeruginosa, Staphylococcus aureus, Enterococcus faecalis*	Root bark extracts showed good activity in all test strains (0.31 ‐ 1.25 mg/mL). Stem bark extracts exhibited good activity with MIC values of 0.63 ‐ 1.25 mg/mL. The leaf extracts were least active against *K. pneumoniae* and *E. faecalis* (MIC = 2.50 mg/mL), and highly active against the other test strains (MIC = 0.31 ‐ 0.63 mg/mL).	[180]
*Colophospermum mopane* (J. Kirk ex Benth.) J. Léonard	Bark	Water, methanol	Microplate serial dilution	*Staphylococcus aureus, Escherichia coli*	Water (7.71 mg/mL) and methanol (5.99 mg/mL) extracts were active against *S*. *aureus* and *E. coli* (12.1 mg/mL and 7.86 mg/mL, respectively). Control: Gentamycin and Ampicillin	[181]
*Colophospermum mopane* (J.Kirk ex Benth.) J.Léonard	Bark, leaves	Water, ethanol	Disc agar diffusion assay	*Staphylococcus aureus, Pseudomonas aeruginosa, Candida albicans*	Leaf (20 mg/mL) and bark water extract (5 mg/mL &10 mg/mL) were effective against *S. aureus* and *P. aeruginosa*, respectively. All ethanol extracts were active against the tested bacterial strains. Control: Penicillin G	[172]
*Combretum hereroense* Schinz	Leaves	Methanol	Microplate serial dilution	*Bacillus cereus, Escherichia coli, Klebsiella pneumoniae, Salmonella typhimurium, Staphylococcus aureus, Enterococcus faecalis, Pseudomonas aeruginosa*	The extracts were active against all the test strains, with an average MIC of >1.75 mg/mL. Control: Ciprofloxacin (0.01 mg/mL)	[155]
*Combretum hereroense* Schinz	Leaves	Acetone, hexane, DCM, methanol	Microplate serial dilution	*Candida albicans, Cryptococcus neoformans, Aspergillus fumigates, Sporothrix schenkii, Microsporum cannis*	All extracts were highly active against the fungal strains after 24 and 48‐h periods. Extract activity was calculated at 0.39 mg/mL (acetone), 0.6 mg/mL (hexane), 0.67 mg/mL (DCM) and 0.24 mg/mL(methanol). Control: Amphotericin B	[182]
*Combretum imberbe* (Wawra)	Leaves	Acetone, hexane, DCM, methanol	Microplate serial dilution	*Candida albicans, Cryptococcus neoformans, Aspergilllus fumigates, Sporothrix schenkii, Microsporum cannis*	The extracts were active against all the test fungal strains after 24 and 48‐h period. Acetone and methanol extracts were active against *C. albicans* (>2.5 mg/mL) while DCM, acetone and hexane extracts were active against *S. schenkii* (2.5 mg/mL). Control: Amphotericin B	[182]
*Combretum imberbe* (Wawra)	Leaves	Methanol	Microplate serial dilution	*Bacillus cereus, Escherichia coli, Klebsiella pneumoniae, Salmonella typhimurium, Staphylococcus aureus, Enterococcus faecalis, Pseudomonas aeruginosa, Staphylococcus epidermidis*	The extracts were active against all the test strains, with an average MIC = 0.24 mg/mL. Control: Ciprofloxacin (0.01 mg/mL)	[155]
*Croton gratissimus* Burch*. *	Leaves	Methanol	Microplate serial dilution Agar disc diffusion assay	*Staphylococcus aureus, Staphylococcus epidermis, Staphylococcus aureus*	The highest minimum inhibition zone of extracts was observed against for *S. aureus* (20 mm) and hospital isolate *S. epidermidis* (27 mm). Control: Cloxacillin	[86]
*Croton gratissimus* Burch.	Leaves	Ethanol	Microplate serial dilution	*Candida albicans, Mycobacterium aurum*	Extracts were effective against *Mycobacterium aurum* (2.5 mg/mL) and *C. albicans* (3.5 mg/mL). Control: Fungazole and Ampicillin	[90]
*Dicerocaryum eriocarpum* (Decne.) Abels (Syn: *Sesamum eriocarpum (*Decne.) Byng & Christenh.)	Roots	Ethanol	Microplate serial dilution	*Candida albicans, Mycobacterium aurum*	Root extracts were active against *C. albicans* (4.5 mg/mL) and *Mycobacterium aurum* (0.156 mg/mL). Control: Fungazole and Ampicillin	[90]
*Dichrostachys cinerea* (L.) Wight & Arn*. *	Roots	Ethanol	Microplate serial dilution	*Candida albicans, Mycobacterium aurum*	The extract showed activity against *C. albicans* at 2 mg/mL and *Mycobacterium aurum* at 0.156 mg/mL. Control: Fungazole and Ampicillin	[90]
*Diospyros lycioides* Desf.	Leaves	Hexane, acetone, ethyl acetate, methanol	Bioautography	*Pseudomonas aeruginosa, Staphylococcus aureus, Enterococcus faecalis*	Ethyl acetate and acetone extracts were active against across the test strains. Whereas the methanol and hexane extracts exhibited antibacterial activity against *S. aureu*s and *E. faecalis* as well as *E. faecalis*, respectively.	[130]
*Drimia sanguinea* (Schinz.) Jessop	Bulb	Methanol, petroleum ether	Microplate serial dilution	*Bacillus cereus, Candida albicans, Candida glabrata, Trichophyton tomsurans*	The MIC value for methanol extracts against *Candida albicans* was the lowest at 1.56 mg/mL.	[88]
*Elephantorrhiza elephantina* (Burch) Skeels	Roots	Ethanol	Microplate serial dilutions	*Candida albicans, Bacillus cereus, Escherichia coli, Staphylococcus aureus, Pseudomonas aeruginosa, Mycobacterium aurum*	The extracts were active against all the test strains, with the highest MIC value of 3 mg/mL. Control: Fungazole and Ampicillin	[90]
*Elephantorrhiza elephantina* (Burch) Skeels	Rhizomes	Methanol, petroleum ether	Microplate serial dilution	*Bacillus cereus, Candida albicans, Trichophyton tonsurans*	Methanol extracts showed activity against *B. cereus* (20 mg/mL), *Candida albicans* (20 mg/mL), and *T. tonsuran*s (10 mg/mL). Controls: Neomycin (antibacterial) and Amphotericin B (antifungal)	[88]
*Euphorbia serpens* Kunth	Shoots, roots	Ethanol, methanol, DCM, petroleum ether	Disc diffusion assay	*Salmonella typhi, Streptococcus pneumoniae, Enterococcus faecalis, Aspergillus fumigatus*, *Candida albicans, Fusarium oxysporum*	Ethanol extracts showed the strongest activity, except with the inhibition of *E. faecalis, F. oxysporum* and *S. pneumoniae*. Petroleum ether extracts were mainly effective against *Candida albicans*.	[183]
*Gomphocarpus fruticosus* (L.) W.T.Aiton	Aerial parts, fruits	Hexane, methanol	Microplate serial dilution assay	*Staphylococcus aureusm Enterococcus faecalis, Klebsiella pneumoniae, Mycobacterium smegmatis*	The antibacterial activity for aerial extracts was >250 µg/mL. Hexane fruit extracts had good activity against *E. faecalis* (125 µg/mL) and *P. aeruginosa* (31 µg/mL). Controls: Gentamicin, Rifampicin, Vancomycin	[184]
*Grewia flava* DC*. *	Twig, roots	Hexane, acetone, distilled water	Agar well diffusion method Microplate serial dilution assay	*Pseudomonam aeruginosa, Staphylococcus aureus, Escherichia coli*	Microbial inhibition from all the extracts was positive against all the test strains. Control: Chloramphenicol	[185]
*Grewia flava* DC.	Roots	Acetone	Bioautography Microplate serial dilution assay	*Candida albicans, Cryptococcus neoformans, Staphylococcus aureus, Proteus mirabilis, Moraxella catarrhalis, Klebsiella pneumoniae* *Bacillus cereus, Proteus vulgaris, Mycobacterium smegmatis, Mycoplasma hominis, Escherichia coli, Pseudomonas aeruginosa*	Acetone extracts had the lowest average MIC value (247 µg/mL), hexane extracts have the highest average MIC value (923 µg/mL). Control: Amphotericin B, Vancomycin and Strepromycin	[51]
*Helichrysum paronychioides* DC*. *	Whole plant	Methanol, petroleum ether	Microplate serial dilution	*Bacillus cereus, Candida albicans, Trichophyton tonsurans*	The methanol extract had noteworthy antimicrobial activity against *B. cereus* and *T. tonsuran*s (0.39 mg/mL). Petroleum ether extracts also exhibited good activity against *S, flexneri* (0.1 mg/mL). Controls: Neomycin (antibacterial) and Amphotericin B (antifungal)	[88]
*Jatropha zeyheri* Sond	Roots Leaves	Acetone, methanol, ethyl acetate	Microplate serial dilution assay	*Escherichia coli, Pseudomonam aeruginosa, Enterobacter cloacae, Klebsiella pneumoniae, Serrattia marscens, Samonella spp., Staphylococcus aureus, Bacillus cereus, Bacillus pumilus*	The extracts had clear inhibition zones for the test strains indicating activity. Methanol leaf extracts showed poor activity (>12.5 mg/mL) against *E. coli* and *P. aeruginosa*, similar to the activity of acetone leaf extract against *S. marscen*s. Methanol root extracts also have the same activity MIC value (>12.5 mg/mL) against *S. aureus, B. pumilus* and *A. calcaoceutical*. Control: Neomycin, Metronidazole	[51]
*Malva neglecta* Wallr*. *	Leaves	Methanol, water	Disc agar diffusion assay, Well agar diffusion	*Escherichia coli, Klebsiella pneumoniae, Pseudomonas aeruginosa, Proteus mirabilis, Staphylococcus aureus*	Methanol extracts inhibited growth of *S. aureus* and *K. pneumonia*.	[186]
*Malvastrum coromandelianum* (L.) Garcke	Leaves	Methanol	Microplate serial dilution	*Mycobacterium fortuitum, Mycobacterium smegmatis, Mycobacterium aurum*	Extracts inhibited microbial growth of *M. fortuitum* and *Mycobacterium aurum* (0.63 mg/mL) as well as *M. segmantis* (0.31 mg/mL). Control: Streptomycin and Rifampicin	[126]
*Malvastrum coromandelianum* (L.) Garcke				*Candida albicans *	Ethyl acetate extract at 10 µg/mL inhibited *Candida albicans* (8 mm) while ethanol extract at 10 µg/mL inhibited *Candida albicans* (7 mm). Control: Fluconazole	[13]
*Nicotiana tabacum* L*. *	Leaves	Ethyl acetate	Agar well diffusion assay	*Pseudomonam aeruginosa, Klebsiella pneumonia, Staphylococcus aureus, Salmonella enterica subsp. enteric serotype Typhi, Micrococcus sp., Proteus mirabilis, Klebsiella sp., Escherichia coli*	Extract activity against *S. aureus* was 500 µg/mL, with an inhibition zone of approximately (160 mm).	[187]
*Opuntia ficus‐indica* (L) Mill	Fruits	Ethanol	Disc diffusion assay	*Staphylococcus aureus, Bacillus cereus, Listeria monocytogenes, Escherichia coli, Salmonella typhi*	The inhibition zones of 50 mg/mL were 17 mm against *S. typhi* and 35 mm for *B. cereus*. The lowest tested concentration (3.12 mg/mm) only inhibited *B. cereus* (3.12 mm) Control: Cyclohexane	[186]
*Osyris lanceolata* Hochst. & Steud*. *	Leaves	Methanol	Disc diffusion assay	*Escherichia coli, Staphylococcus aureus*	*S. aureus* inhibition for the smallest tested concentration (1 mg disc) was 6,0 mm. *E. coli* inhibition for the smallest tested concentration (1 mg disc) was 6,17 mm. Ampicillin and Ciprofloxacin	[188]
*Osyris lanceolata* Hochst. & Steud.	Bark	DCM, water, ethyl acetate	Microplate serial dilution	*Proteus mirabilis, Klebsiella pneumonia, Salmonella typhi, Escherichia coli, Pseudomonas aeruginosa*	The extracts showed some activity against the test strains (6.25 and 12.5 mg/mL). Control: Gentamicin	[189]
*Ozoroa paniculosa* var*. paniculosa *	Leaves	Acetone, hexane fractions, DCM, ethanol, butanol	Microplate serial dilution	*Staphylococcus aureus, Pseudomonas aeruginosa, Escherichia coli, Enterococcus faecalis, Aspergilus fumigatus, Candida albicans, Cryptococcus neoformans*	Water extracts activity against fungal strains ranged from 625 ‐ 2500 µg/mL. Extracts exhibited activity (1250 µg/mL) against *E. coli* and *E. faecalis*. The lowest activity (19 µg/mL) was observed in hexane and DCM fractions against *S. albicans* and *E. faecalis*. Control: Amphotericin B (0.78–6.25 µg/mL); gentamicin (0.39–1.56 µg/mL).	[190]
*Peltophorum africanum* Sond*. *	Stem, bark	Ethyl acetate	Well diffusion assay	*Staphylococcus aureus, Pseudomonas aeruginosa, Aeromonas hydrophila, Shigella sonnei, Salmonella typhimurium, Aspergillus flavus, Candida albicans, Cryptococcus neoformans*	The extracts were inactive against *A. flavus* at all tested concentrations. The extracts with MIC values above 2.5 mg/mL were *Candida albicans* (5 mg/mL) and *C*. *neoformans* (10 mg/mL). Control: Tetracycline and Amoxicillin	[189]
*Rhus lancea *L. F.	Leaves	Steam distillation	Disc diffusion assay	*Acinobacter calcoaceticus, Citrobacter freundii, Clostridium perfringens, Clostridium sporogenes, Escherichia coli, Klebsiella pneumoniae, Proteus vulgaris, Pseudomonas aeruginosa, Salmonella typhi, Staphylococcus aureus, Yersinia enterocolitica, Candida albicans, Aspergillus flavus*	Essential oil concentrations tested (10, 20, µg/mL) against *K. pneumonia* were reported to be resistant to the strain. Bacterial strains tested were susceptible to the different essential oil concentrations resulting in inhibition zones between 4.0 – 19.2 mm. Essential oil concentrations of 100 µg/mL had an inhibition zone of 74.2 mm, compared to the 76.2 mm inhibition zone of the control used.	[191]
*Ricinus communis* L.	Leaves	n‐Hexane, chloroform, DCM, ethyl acetate, acetone, ethanol, methanol	Microplate serial dilution TLC‐bioautography	*Staphylococcus aureus, Enterococcus faecalis, Escherichia coli, Pseudomonas aeruginosa*	Hexane extracts showed good activity against the test strains (0.61 mg/mL). The hexane and ethanol extract had the best activity against *P. aureginosa* (0.13 mg/mL). The ethanol extracts showed activity (0.31 mg/mL) against the strains	[192]
*Schkuhria pinnata* (Lam.) Kuntze ex Thell.	Aerial parts	Hexane, DCM, acetone, ethyl acetate	Microplate serial dilution	*Mycobacterium smegmatis*	Extracts inhibited bacterial growth, with acetone extracts having good activity (0.27 mg/mL) compared with hexane extracts (2 mg/mL).	[120]
*Searsia lancea* (L.f.) F.A. Barkley	Leaves	Ethanol, acetone	Microplate serial dilution	*Streptococcus agalactiae, Streptococcus uberis SUB 1 – 7, Streptococcus uberis ATCC 700407, Escherichia coli ECO 1 – 7, Escherichia coli ECO ATCC 25922*	Extracts were effective against the test strains with activity ranging between 0.06 and 0.2 mg/mL.	[93]
*Securidaca longepedunculata* Fresen.	Roots, bark	Ethanol	Disk diffusion assay	*Candida albicans *	The extract was active against *Candida albicans. C. mycoderma* and *P. aeruginosa* (Inhibition zones: 2.0 ± 0.3, 3.0 ± 0.3 & 1.0 ±0.0 mm, respectively). Control: Fungazole and Ampicillin	[90]
*Senna italica subsp. Arachoides* Burch Lock	Roots	Acetone	Microplate serial dilution	*Pseudomonas aeruginosa, Enterococcus faecalis, Escherichia coli, Staphylococcus aureus*	The extract exhibited good activity against the test strains, with an average activity of 0.12 mg/mL.	[193]
*Senna tora* (L.) Roxb*. *	Leaves	Ethyl acetate	Agar well diffusion assay	*Staphylococcus aureus, Enterococcus sp., Escherichia coli, Salmonella typhi, Pseudomonas aeruginosa, Haemophilus influenzae*	Extracts were active against all strains. Extract concentrations of 62.5, 125 mg/mL, 250 mg/mL & 500 mg/mL, had smaller inhibition zones than the control. Control: Erythromycin	[135]
*Tarchonanthus camphoratus* L*. *	Leaves	Hexane, DCM, methanol	Disc diffusion assay	*Escherichia coli, Klebsiella pneumoniae, Pseudomonas aeruginosa, Proteus mirabilis, Salmonella typhi, Staphylococcus aureus, Bacillus spp., Candida albicans*	Screening for activity revealed low inhibition (no or small inhibition zones) at 400 mg/mL concentration. Ethyl acetate extracts exhibited good activity (0.57 mg/mL) against *Bacillus spp*. and methanol extract gave the highest MIC activity against *Candida albicans* (2 mg/mL x 10^2^). Control: Chloramphenicol and Nystatin	[194]
*Terminalia sericea* Burch. Ex DC	Leaves	Acetone, methanol	*In vitro* Serial dilution microplate assay *In vivo* Topical application	*Candida albicans, Cryptococcus neoformans, Microsporum canis, Shigella schenckii, Aspergillus fumigates*	A 2 g/10 g dose (extract suspended in aqueous cream) administered to wounds inhibited infections after inoculation by test fungi. The *in vivo* studies showed good antifungal activity ranging from 0.02 ‐ 0.64 mg/mL. Control: Amphoterecin B	[94, 97]
*Terminalia sericea* Burch. Ex DC	Leaves	Methanol		*Staphylococcus aureus, Bacillus cereus, Staphylococcus epidermis, Enterococcus faecalis, Escherichia coli, Salmonella typhirium, Pseudomonas. aureginosa, Klebsiella pneumonia*	Plant extracts were effective against all tested strains. The MIC values range from 0.6 to >3.0 mg/mL. The average MIC activity against the strains was >1.49 mg/mL.	[155]
*Vachellia karroo* (Hayne) Banfi & Galasso	Leaves, roots	Chloroform, methanol, ethanol and ethyl acetate	Petri dish/ disc diffusion	*Staphylococcus aureus, Escherichia coli, Klebsiella pneumonia, Salmonella typhi, Pseudomonas aeruginosa*	Root extracts showed higher bacterial inhibition against all tested strains.	[195]
*Vachellia nilotica* (L.) P.J.H.Hurter & Mabb	Bark and leaves	Acetone, water	MIC Assay	*Staphylococcus aureus, Streptococcus uberis, Streptococcus agalactiae, Streptococcus chromogenes, Streptococcus epidermidis, Escherichia coli, Klebsiella pneumonia, Pseudomonas aeruginosa, Proteus mirabilis, Enterobacter aerogenes*	Acetone bark extract exhibited strong antibacterial activity against several *Staphylococcus* strains as well as *P. aeruginosa, P. vulgaris* and *E. aerogenes* strains. Control: Gentamycin	[134]
*Ximenia americana* L.	Bark, leaves, roots, stem	Water, methanol, chloroform	Cup‐plate agar diffusion, agar dilution method	*Pseudomonas aeruginosa, Escherichia coli, Staphylococcus aureus, Candida albicans*,	All methanol extracts had poor activity against the tested bacteria, MIC value range 0.21 ‐ > 72.75 mg/mL. S. aureus was most susceptible to methanol bark 0.62 mg/mL), water bark (1.62 mg/mL), methanol leaf (0.77 mg/mL) and water leaf extract (MIC = 1.99 mg/mL) Control: ampicillin, benzyl penicillin, cloxacillin, gentamicin (5, 10, 20 & 40 µg/mL). Methanol/water extracts (leaf, root, stem) were ineffective against *Candida albicans*. Controls: Clotrimazole (5 µg/mL) and Nystatin (25 µg/mL)	[158]
*Ziziphus mucronata* Willd. subsp*. mucro nata *	Leaves	Ethanol	Microplate serial dilution, Agar disc diffusion	*Mycobacterium aurum, Bacillus cereus*	Extracts were active against *Mycobacterium aurum* (0.625 mg/mL), and *B. cereus* (2 mg/mL). Control: Ampicillin	[90]
*Ziziphus mucronata *	Leaves	n‐Hexane, chloroform, DCM, ethyl acetate, acetone, ethanol, methanol	Microplate serial dilution, TLC‐bioautography	*Staphylococcus aureus, Enterococcus faecalis, Escherichia coli, Pseudomonas aeruginosa*	Hexane extracts showed good activity, the lowest average MIC value (0.50 mg/mL). hexane extracts had the best activity against *E. coli* (MIC = ). The highest activity (MIC = 1.05 mg/mL) for acetone and methanol extracts was against *E. faecalis*. Control: Not specified	[192]

**Abbreviations**: Bw, body weight; DCM, dichloromethane; MIC, minimum inhibitory concentration; TCM, trichloromethane; TLC, thin layer chromatography

**TABLE 5:** Summary of analysis of anti‐parasitic activity of plants used by Batswana for livestock health management.

Plant species	Plant part(s)	Extraction solvent	Bioassay and Application	Parasite species tested	Summary of findings	References
*Acokanthera oppositifolia (*Lam.) Codd	Leaves, twigs	Petroleum ether, DCM, ethanol, water	*In vitro* colourmetric assay	*Caenorhabditis elegans *	Petroleum ether and ethanol leaf extracts had high anthelmintic activity (MIC = 0.52 mg/mL) Control: 1 g/1 mL Levamisole	[122]
*Aloe ferox* Mill*. *	Leaves	Water	*In vivo* oral administration	*Heterakis gallinarum *	All the tested doses (50, 100, 200 and 400 mg/kg) reduced worm egg count (*in vivo*) after 14 days, with 100 and 400 mg/kg plant extracts exhibiting 99 and 100% egg count reduction. The 200 mg/kg dose resulted in the highest worm count reduction (± 85%) after slaughter. Control: Mebendazole	[108]
*Aloe ferox* Mill*. *	Leaves	Water	*In vitro* egg hatch assay Larval Development assay Larval mortality assay	*Haemonchus contortus *	All tested concentrations showed positive anthelmintic activity. The 7.5 mg/mL extract had the highest egg hatch inhibition (± 45%) and larval development inhibition percentage of ± 95%`. All tested concentrations (2.5, 5.0 & 7.5 mg/mL) exhibited high larval mortality concentration (99.1, 100 & 100 %) after 72 h. Control: 2.5, 5.0 & 7.5 mg/mL Thiambedazole	[196]
*Aloe ferox* Mill*. *	Leaves	Water (hot)	Egg hatch assay Larval development assay	*Haemonchus contortus *	Good egg hatch inhibition from concentration of 2.5 mg/mL (807%) onwards to 100% inhibition was achieved with 20 mg/mL extract (maximum concentration tested). The 1.25 mg/mL and 0.625 mg/mL extracts had the lowest inhibition (33.1% and 0.48%, respectively). Good larval development inhibition (98.9%) observed at the lowest tested concentration of 0.625 mg/mL. Control: Albendazole	[54]
*Aloe marlothii* A. Berger	Leaves	Acetone		*Ehrlichia ruminantium *	Plant extract with concentration of 50 mg/mL was effective against *E. ruminantium *	[197]
*Amaranthus blitum* subsp*. oleraceus (*L.) Costea	Aerial parts	Ethanol	*In vitro* larvicidal assay	*Trichinella spiralis *	Extracts exhibited strong larvicidal activity at concentration of 1 mg/mL after 72 h, results show a 15% larval viability. Control: 20 µg/mL ABZ	[198]
*Artemisia afra* Jacq. ex Willd.	Leaves	Water (hot)	Adult worm inhibition	*Schistosomula mansoni *	Hexane and DCM extracts (100 µg/mL) inhibited 100% of the adult worms after 24, 48 and 72 h with a final IC_50_ value of 10.4 µg/mL (hexane) and 8.9 µg/mL (DCM) after 72 h. Control: praziquantel	[199]
*Combretum hereroense* Schinz	Leaves	Ethyl acetate, acetone, water	Microplate antischistosomal assay	*Schistosoma haematobium*, *Caenorhabditis elegans* var. *Bristol*	Acetone (80%–90% viability) and ethyl acetate (70%–80% viability) extracts were active against *C. elegans* at a concentration of 1 mg/mL. Water extracts were tested for antischistomosal activity, with no activity reported.	[200]
*Dicerocaryum eriocarpum (*Decne*.)* Abels (Syn*: Sesamum eriocarpum (*Decne*.)* Byng & Christenh*.)*	Leaves	Water	Egg hatch assay Larval development assay	*Haemonchus contortus *	The tested concentrations (2.5, 5, 7.5 mg/mL) were active against the tested parasite. The 7.5 mg/mL extract inhibited 100% egg hatching, 5 mg/mL and 7.5 mg/mL concentrations exhibited 100% larval development inhibition. Control: 2.5, 5.0 & 7.5 mg/mL Thiambedazole	[196]
*Nicotiana tabacum* L*. *	Leaves	Methanol	*L. sericata* development test *Caenorhabditis elegans* development test *Ctenocephalides felis* adult oral test *Ctenocephalides felis* adult contact test, Tick repellent/ knockdown contact test	*Ctenocephalides felis* *Lucilia seriata* *Caenorhabditis elegans* *Rhipicephalus sanguineus (*adult) *Rhipicephalus sanguineus (*larvae) *Ixodes Ricinu*	Minimum effective concentration for *L. sericata* larvae was >65 µg/mL, for *C. felis* concentration activity was >565 mg/mL, and for the percentage oral activity it was > 200 µg/mL. Controls: hiamethoxam, dicyclanil, ivermectin, and *N, N*‐diethyl‐m‐toluamide (DEET)	[110]
*Sclerocarya birrea* Hochst*. *	Bark	Methanol, water (cold/hot)	Larval mortality assay	*Haemonchus, Oesophagostomum Trichostrongylus*	Methanol extracts were effective against parasites.	[201]
*Securidaca longepedunculata* Fresen*. *	Roots	Water‐ethanol	*In vitro* mortality assay *In vivo* Postmortem worm count Faecal egg count	*Haemonchus contortus* *Heligmosomoides polygyrus*	*In vitro* The extracts exhibited 75% mortality in the tested species (*H. contortus*), and 70% mortality of *H. polygyru*s. Control: Levamisole *In vivo* Worm count decreased by 80% after 500 mg/kg b.w. dose, worm count decreased by 88% after 1000 mg/kg b.w. (course duration: 4 days) Feacal egg count was increased after 4 consecutive days of treatment Control: Pyrantel embonate	[202]
*Vachellia nilotica* (L.) P.J.H. Hurter & Mabb	Stem bark	Water	Trypanocidal activity Low Inoculation Long Incubation Test (LILIT) Long‐Term Viability Assay (LtVA)	*Trypanosoma brucei brucei STIB 345 *	Extracts had in vitro trypanocidal activity (MIC = 5 µg/mL) Stem extracts showed poor *in vivo* activity (MIC = 100 µg/mL) when 100–150 mg/kg body weight dose was administered orally. Control agent: diminazene aceturate (Berenil)	[203]
*Ximenia americana* L. var. *microphylla *Welw. ex Oliv.	Bark	Hydroethanol	Acaricidal adult immersion assay	*Rhipicephalus microplus *	Bark extract concentrations of 5 ‐ 80 mg/mL had acaricidal mortalities ranging from 13 ‐ 100%.	[204]
*Thamnosma rhodesica* (Baker f.) Mendonça	Roots	DCM	TLC‐Bioautography Antileishmanial assay	*Leishmania major*	Minimum amount for inhibiting fungal growth is 10 µg/mL. Control: Niastatin. Plant extracts inhibit reduce population of free‐living parasites (survival rate range from 30%–90%) and intracellular parasite (survival rate range 6.2% – 917 %). Control: Amphoterecin B	[205]

Abbreviations: b.w, body weight; DCM, dichloromethane; MIC, minimum inhibitory concentration; TLC, thin layer chromatography.

**TABLE 6:** Summary of inflammation and pain study results of plants used by Batswana for livestock health management.

Plant species	Plant part(s)	Extraction solvent	Biological assay	Summary of findings	References
*Acokanthera oppositifoli*a (Lam.) Codd	Leaves, twigs	Petroleum ether, DCM, ethanol, water	COX 1 and 2	Leaf petroleum ether, DCM and ethanolic extract showed good COX‐1 inhibition (98, 99, 97%). Leaf petroleum ether and DCM extracts exhibited good COX‐2 inhibition (85, 81%).	[122]
*Aloe ferox* Mill.	Leaves	Water		Extracts exhibited anti‐inflammatory and analgesic activities at a dose extract of 400 mg/kg.	[108]
*Bulbine abyssinica *	Whole plant	Acetone, water	Protein denaturation method	Anti‐inflammatory activity of all extracts was observed 200 µg/mL dose.	[206]
*Combretum hereroense* Schinz	Leaves	Ethyl acetate, acetone, water		Plant extract exhibit *in vitro* inflammatory reaction at concentrations of 0.5 and 1 mg/ mL	[200]
*Malvastrum coromandelianum* (L.) Garcke	Leaves	Methanol	15‐LOX	Extracts and fractions were inhibitory to 15‐LOX, IC_50_ for the crude extract is 77.52 ±1.31	[207]
*Nicotiana tabacum* L.	Seeds	Phytosterol isolates	COX 1 and 2	Extract demonstrated inhibition of COX‐2 by down‐regulate the expression of COX‐2 mRNA and were ineffective against the expression COX‐1 mRNA.	[116]
*Ozoroa paniculosa* var*. paniculosa *	Leaves	Acetone	15‐LOX	Maximum inhibition of >50% was observed when tested at 0.128 mg/mL extract concentration.	[208]
*Portulaca oleracea* L.	Aerial parts	Ethanol	Hind paw volume, reduction in cotton pellet weight, intraperitoneal administration	Time dependent positive anti‐inflammatory activity after 400 mg/kg dose, maximum hind paw volume increase of 67.1 ± 6.18 % after 240 mins. Percentage increase in cotton pellet weights in the control (86.69 ± 11.8%) after intraperitoneal administration. Control: diclofenac 4 mg/kg	[209]
*Senna tora* (L.) *Roxb*.	Leaves	Ethyl acetate	Heat induced haemolysis of RBC assay, BSA protein denaturation assay	Inhibition of RBC membrane hemolysis range of 31.058 ± 3.145% to 89.029 ± 1.186%, (IC_50_ = 28.309 µg/mL), tested dosage: 20 – 220 µg/mL. BSA denaturation inhibition range of 32.617 ± 0.890% to 91.731 ± 0.949% (IC_50_ = 22.980 µg/mL), tested dosage: 20–220 µg/mL. Control: ibuprofen 20–220 µg/mL.	[135]
*Terminalia sericea* Burch. Ex DC	Leaves	Acetone, methanol	*In vivo*, topical application	Wound healing activity observed is indicative of in vitro anti‐inflammatory activity (2 g/10 g dose)	[94, 97]
*Vachellia karroo* (Hayne) Banfi & Galasso	Bark	Water	*In vivo*, administered via injection	Extracts had good analgesic and anti‐inflammatory activity in mice at doses of 100 and 200 mg/kg, comparable to the control used Control: Indomethacin (10 mg/kg)	[123]
*Vachellia tortilis* (Forssk.) Galasso & Banfi	Seeds	Water	*In vivo* formalin induced paw lick test, acetic acid induces writhing test, hot plate test, tail flick method	Anti‐nociceptive activity (rats) when 100 and 200 mg/kg body weight administered orally Control agents: Morphine sulphate + Naloxone, Morphine sulphate, Diclorofenac sodium	[117]

Abbreviations: BSA, bovine serum albumin; COX, cyclooxygenase; DCM, dichlorormethane; LOX, 15‐lipoxygenae; NO, nitric acid; RBC, red blood cells.

**TABLE 7:** Summary of reported antioxidant activity of plants used by Batswana for livestock health management.

Plant species	Plant part(s)	Extraction solvent	Biological assay	Summary of findings	References
*Acokanthera oppositifolia* (Lam.) Codd	Stem	Methanol	ABTS inhibition, DPPH scavenging	Extracts showed good ABTS inhibition (80 % inhibition) at concentration of 0.02 mg/mL. DPPH inhibition at 0.02 mg/mL was between 50%–60%.	[125]
*Acokanthera oppositifoli*a (Lam.) Codd	Leaves	Acetone, ethyl acetate, chloroform, hexane, water	NO inhibition	*In vitro* analysis showed no NO‐inhibition at 10 mg/mL.	[126]
*Acokanthera oppositifoli*a (Lam.) Codd	Leaves	Acetone, chloroform,, methanol	Hydroxyl free radical scavenging, Superoxide anion scavenging, Fe (III) reduction	Methanol extracts exhibited better Fe^3+^ reducing ability (IC_50_ = 0.234 mg/mL) than acetone (IC_50_ = 0.22 mg/mL) and chloroform (IC_50_ = 0.242 mg/mL). Superoxide anion scavenging activity was ± 78% for methanol extracts, ±66% for chloroform extracts and ±60% for acetone extracts. Acetone and chloroform extracts inhibit ±83% and methanol exhibit ±75% hydroxyl scavenging activity.	[17]
*Aloe barbadensis* Mill.	Leaves	Methanol	ORAC scavenging, HPS Scavenging, DPPH Scavenging	Extracts were found to have ORAC scavenging activity (TE ≈700 µmol/g), DPPH scavenging activity (TE ≈36 µmol/g), and HPS scavenging activity (TE ≈ 17 µmol/g).	[210]
*Aloe ferox* Mill.	Leaves	Methanol	DPPH scavenging, ABTS inhibition	Extracts reached 60% DPPH and 80% ABTS inhibition at 0.4 mg/mL.	[129]
*Aloe ferox* Mill.	Leaves	Methanol	ORAC scavenging, HPS Scavenging, DPPH Scavenging	Extracts exhibited ORAC scavenging activity (TE≈1000 µmol/g), DPPH scavenging activity (TE≈34 µmol/g), and HPS scavenging activity (TE≈37 µmol/g).	[210]
*Aloe marlothii* Mill.	Leaves	Methanol	ORAC scavenging, HPS Scavenging, DPPH Scavenging	Extracts showed ORAC, DPPH and HPS scavenging activity of ±1500 µmol/g, ± 55 mol/g, and ± 42 µmol/g, respectively.	[211]
*Aloe marlothii* A. Berger	Leaves	Methanol	DPPH scavenging	Extract concentration of 5 g/40 mL had DPPH scavenging activity of ±55 µmol/g.	[211]
*Asparagus laricinus *	Stem, leaves	Water	DPPH scavenging	Leaf and stem extract (2.5 mg/mL) exhibited 72% and 63% scavenging activity, respectively.	[135]
*Cassia abbreviata* Oliv.	Roots	Methanol	*In vivo* oral administration	Dosages of 200 and 500 mL/kg reduced oxidative stress in chickens and increased liver enzymes.	[137]
*Diospyros lycioides*	Leaves	Acetone, ethyl acetate, hexane, methanol	DPPH scavenging	Chromatograms elute in CEF had the highest number of compounds that with good DPPH scavenging activity; methanol extract had 4 bands, acetone extracts had 6 bands, ethyl acetate extract had 4 bands and hexane extracts had 1 band. The hexane extracts on the EMW eluted plate had no visible bands. All the extracts had 1 band on the BEA eluted plate, with similar R_f_ values (0.9 – 0.96).	[130]
*Drimia sanguinea* (Schinz.) Jessop	Bulb	Methanol	DPPH scavenging, B‐carotene linoleic acid	The extracts exhibited 64% DPPH scavenging activity (EC_50_ = 92.6 µg/mL)	[88]
*Elephantorrhiza elephantina*	Rhizomes	Methanol	DPPH scavenging	Extracts exhibited 84.7% antioxidant activity (EC_50_ = 5.8 µg/mL)	[88]
*Euphorbia serpens* Kunth	Shoots, roots	DCM, ethanol, methanol, petroleum ether	DPPH scavenging	All extracts exhibit antioxidant activity. Methanol shoot extracts had an IC_50_ value of 13.17 µg/mL, and DCM shoot extract had the highest IC_50_ value (553.26 µg/mL).	[130]
*Grewia flava* DC.	Twig, roots	Hexane, acetone, distilled water, methanol	DPPH scavenging, Ferric reducing, Metal Chelating	Methanol twig extracts were the most active at an IC_50_ of 14.50 µg/mL for DPPH scavenging activity, and distilled water twig extract had an IC_50_ value of 495 µg/mL. Metal chelation IC_50_ values were above 100 µg/mL, and ferric reducing power ranged from 637 ‐ 745 mg AAE/g.	[185]
*Helichrysum paronychioides* DC.	Whole plant	Methanol	DPPH scavenging	The extract exhibited 84.4% DPPH scavenging activity (EC_50_ = 20.1 µg/mL)	[88]
*Malva neglecta* Wallr.	Whole plant	Water‐methanol (30:70)	DPPH scavenging	The tested extract concentrations (0.0156 ‐ 1 mg/mL) exhibited antioxidant activity which increased depending on the concentration tested. The highest inhibition (70%) was observed at 1 mg/mL.	[212]
*Ipomoea oblongata* E. Mey. ex Choisy	Roots	Water, methanol, DCM	DPPH scavenging	The extract showed DDPH scavenging activity of 98% at 0.5 mg/mL tested extract concentration.	[213]
*Moringa oleifera* Lam.	Leaves	Methanol, fractions, diethyl ether, chloroform, ethyl acetate, water	*In vitro* DPPH scavenging, OH scavenging, NADH scavenging *In vivo* Oral ingestions, Rat liver dissection	*In vitro* Extract had 80% antioxidant activity; water residue had the lowest antioxidant activity of 22%. Crude extract showed strong reducing power (0.67 ASE/mL) and good DPPH scavenging activity (IC_50_ = 0.122 mg/mL). *In vivo* Extract had good ferrous ion chelating activity (0.45 mg/mL).	[132]
*Ozoroa paniculosa* var. paniculosa	Leaves	Acetone, fractions, hexane, DCM, ethanol, butanol, water	DPPH scavenging, ABTS Inhibition, OH‐ Scavenging	Extracts and ethanol fractions had the best DPPH scavenging activity (EC_50_ = 0.90 and 0.084 µg/mL), and water fractions had poor DPPH scavenging activity (EC_50_ = 663.47 µg/mL). Crude and ethanol extracts had good ABTS scavenging activity (EC_50_ = 0.99 and 1.60 µg/mL).	[190]
*Portulaca oleracea *L.	Whole plant	Water, ethanol	DPPH scavenging, ABTS inhibition	Antiradical activity for water extract showed an IC_50_ of 1.45 mg/g, while the 50% ethanol extract had an IC_50_ value of 0.36 mg/g.	[214]
*Ricinus communis* L.	Leaves	n‐hexane, chloroform, DCM, ethyl acetate, acetone, ethanol, methanol	DPPH scavenging	All extracts showed antioxidant activity on EMW chromatograms.	[192]
*Senna tora* (L.) Roxb.	Leaves	Ethyl acetate	DPPH scavenging, H_2_O_2_ scavenging	Extracts exhibited DPPH (33 ‐ 91%) and H_2_O_2_ (39 ‐ 99%) scavenging activity.	[139]
*Tribulus terrestris* L.	Fruit	Ethanol	NO inhibition	The extracts inhibited NO production at all test concentrations (50, 100, 200 µg/mL).	[153]
*Vachellia karroo (*Hayne) Banfi & Galasso	Leaves	Methanol‐water	ABTS^+^ scavenging, DPPH scavenging	The extracts demonstrated high DPPH scavenging activity (IC_50_ = 4.94 µg/mL) and ABTS^+^ (IC_50_ = 2.23 µg/mL) activity, with moderate FRAP scavenging activity (IC_50_ = 28 µg/mL), further supported by the TLC analysis and NMR.	[121]
*Vachellia nilotica* (L.) P.J.H. Hurter & Mabb	Leaves	Methanol‐water	DPPH scavenging	Extracts at 100 and 200 mg/mL exhibited DPPH scavenging activity.	[215]
*Vachellia tortilis* (Forssk.) Galasso & Banfi	Leaves, trunk bark	Methanol	DPPH scavenging	Leaf and trunk extract had good antioxidant activity (IC_50_ = 0.03 and 0.01 µg/mL).	[120]
*Vachellia tortilis* (Forssk.) Galasso & Banfi	Leaves	Methanol	DPPH scavenging	Low DPPH (IC_50_ = 70 µg/mL), ABTS + (IC_50_ = 52 µg/mL) and FRAP (IC_50_ = 97 µg/mL) scavenging activity was observed in extracts, further supported by the TLC analysis and NMR.	[121]
*Ximenia americana* L. *var. microphylla* Welwe. Ex Oliv.	Fruit, seeds	Ethanol	DPPH scavenging	Seeds showed significant DPPH radical scavenging activity at 200 µg/mL concentration. Both the red and yellow fruit flesh exhibited more than 90% of DPPH free radical scavenging activity at 200 µg/mL concentration.	[149]
*Ximenia americana* L.	Leaves	Methanol	DPPH scavenging	Extracts have a positive antioxidant activity, RC_50_ = 82.50 µg/mL	[216]
*Ximenia americana* L.	Pulp, seeds	Ethanol	DPPH scavenging	The red and yellow pulp extract had DPPH scavenging capacity percentage equal to/more than the ascorbic acid at the tested concentrations (160 and 200 µg/mL). The IC_50_ value for the yellow flesh was 102 µg/mL, and for the yellow seed it was 154 µg/mL.	[149]
*Ziziphus mucronata*	Leaves	n‐hexane, chloroform, DCM, ethyl acetate, acetone, ethanol, methanol	DPPH scavenging	Methanol extracts showed positive antioxidant activity on EMW chromatograms	[192]

**Abbreviations**: ABTS+, 2,2'‐azino‐bis(3‐ethylbenzothiazoline‐6‐sulfonic acid); BEA = benzene: ethanol: ammonium hydroxide; CEF, chloroformethylacetate: formic acid; DCM, dichloromethane; DPPH, 2,2‐diphenyl‐1‐picrylhydrazyl; EMW, ethylacetate: methanol: water; FRAP, ferric reducing antioxidant power; H2O2, dihydrogen peroxide; HPS, NADH, nicotinamide adenine dinuclueotide hydrogen; NMR, nuclear magnetic resonance; NO, nitrous oxide; ORAC, oxygen radical absorbance capacity; TLC, thin layer chromatography.

**TABLE 8:** Summary of results from toxicity and safety studies of plants used by Batswana for livestock health management.

Plant species	Plant part(s)	Extraction solvent	Summary of findings	References
*Asparagus africanus* Lam.	Roots	Water	In vivo No acute oral toxicity was observed on all tested concentrations	[217]
*Burkea africana* Hook.	Stem bark	Ethanol	In vitro Extracts did not show oral toxicity at all tested concentrations. In vivo Extracts had a sedative effect at 2000 mg/kg.	[138]
*Burkea africana* Hook.	Stem bark	Ethanol	In vitro No cytotoxicity observed at 2000 mg/kg.	[138]
*Combretum imberbe* (Wawra)	Leaves	Acetone	In vivo The 10% extracts exhibited cytotoxicity and acute toxicity.	[94, 97]
*Cassia abbreviata* Oliv.	Stem bark	Methanol	In vivo The 200 and 500 mL/kg extract exhibited hepatotoxicity in indigenous chickens.	[137]
*Combretum imberbe (*Wawra)	Leaves	Acetone	In vitro Extracts showed some toxicity, with LC_50_ values ranging from 75.7–168.6 µg/mL ON Vero monkey cells	[94, 97]
*Cassine transvaalensis* (Burtt Davy) Codd Syn: *Elaeondedron transvaalense* (Burtt Davy) R.H. Archer	Stem bark	Ethanol	In vitro Isolated compounds inhibited the growth of Vero cell lines.	[218]
*Drimia sanguinea* (Schinz.) Jessop	Bulb	Methanol, petroleum ether	In vitro Methanol extracts showed toxicity against Vero cells (LC_50_ = 0.015±0.01 µg/mL), and petroleum ether extracts had demonstrated low cytotoxicity (LC_50_ = 552.4± 48 µg/mL).	[88]
*Elephantorrhiza elephantina* (Burch) Skeels	Rhizome	Methanol, petroleum ether	In vitro Methanol extracts were toxic towards Vero cells (LC_50_ = 9.4±3.9 µg/mL) with petroleum ether extracts also showing some level of cytotoxicity with LC_50_ values of 173.4± 13 µg/mL.	[88]
*Gomphocarpus fruticosus* (L.) W.T. Aiton	Aerial parts	Methanol, DCM, chloroform, water	In vivo Acute oral toxicity of extracts had 0% mortality rate for 0.1 g/kg dose, 50% mortality rate for 0.2 g/kg dose, and the highest mortality rate was 0.3 and 0.35 g/kg doses with 83% mortality rate.	[219]
*Helichrysum paronychioides* DC.	Whole plant	Methanol, petroleum ether	In vitro Methanol and petroleum ether extracts exhibited low toxicity against Vero cells (LC_50_ = 24.6±0.4 and 50.2±1.8 µg/mL, respectively).	[88]
*Malvastrum coromandelianum* (L.) Garcke	Leaves	Methanol	In vitro Isolated compounds exhibited low cytotoxicity towards Vero cells.	[126]
*Nicotiana tabacum* L.	Seeds	Methanol, NaOH, HCl	In vivo Isolated compounds were found to be non‐toxic in mice.	[116]
*Nicotiana tabacum* L.	Leaves	Methanol, hexane	In vivo No signs of sub‐acute toxicity were observed on mammalian models (rats).	[140]
*Ozoroa paniculosa* var*. paniculosa *	Leaves	Acetone	In vitro There was some evidence of cytotoxicity (LC_50_ = 16.58 µg/mL) when extracts were studied against Vero cells.	[190]
*Portulaca oleracea* L.	Whole plants	Water, ethanol	In vitro Highest NCTC clone 929 cell viability (124.48%) after 72‐hour incubation with 100 µg/mL polysaccharide fraction, cell viability decreases sequentially with increase in tested concentration (250, 350, 500, 750, 1000 &1500 µg/mL)	[214]
*Ricinus communis* L.	Leaves	n‐Hexane, hexane, chloroform, DCM ethyl acetate, acetone, ethanol, methanol	In vitro No observed toxicity on Vero kidney cells (LC_50_ = 131.8 µg/mL).	[192]
*Securidaca longepedunculata* Fresen.	Roots	Methanol	In vitro Brine shrimp assay revealed a dose dependent increase in mortality, LC_50_ = 74 µg/mL.	[202]
*Senna tora* (L.) Roxb.	Leaves	Ethyl acetate	In vitro Moderately cytotoxic (LC_50_ = 35.246 µg/mL) was observed towards brine shrimp. In vivo Low acute oral toxicity (LD_50_ = 4263.906 mg/kg b. w.), body weight measurement does not indicate acute toxicity	[135]
*Terminalia sericea* Burch. Ex DC	Leaves	Acetone, methanol	In vitro Extracts exhibited low toxicity (LC_50_ = 75.7–168.6 µg/mL) when tested against Vero kidney cells.	[94, 97]
*Thesium* spp.	Fruits	Ethanol	In vitro Cell morphology was not affected, cell viability, cell viability of RAW 264.7 cells >98% after treatment with 50, 100 & 200 µg/mL of extract	[153]
*Vachellia nilotica* (L.) P.J.H.Hurter & Mabb	Bark, leaves	Acetone, water	In vitro Water and acetone extracts exhibit cytotoxicity (LC_50_ = 0.0032 and 0.0278 mg/mL) towards Vero kidney cells	[134]
*Ziziphus mucronata*	Leaves	Acetone, chloroform, DCM, ethanol, ethyl acetate, methanol, n‐hexane	In vitro No observed toxicity towards Vero kidney cells with LC_50_ value of 131.8 µg/mL.	[192]

Abbreviations: b.w., body weight; DCM, dichloromethane; HC, hydrochloric acid; MTT = 3‐ (4,5‐dimethylthiazol‐2‐yl)‐2,5‐diphenyl‐2H‐tetrazolium bromide; NaOH, sodium hydroxide; NCTC, National collection of type cultures

**TABLE 9:** Summary of bioactive phytochemicals and isolated compounds from plants used by Batswana for livestock health management.

Species	Phytochemical analysis	Phytochemicals	Isolated compounds	References
*Acokanthera oppositifolia* (Lam.) Codd	Qualitative chemical assay Quantitative chemical assay (Spectrophotometry)	Flavanols, flavonoids, gallotannins, phenolics, polyphenols, proanthocyanidins, tannins		[17, 122, 126]
*Aloe ferox* Mill.	Quantitative chemical assay (Spectrophotometry), HPLC, GC‐MS	Phenols, flavonoids, flavanols, proanthocyanidins, carotenoids	Lutein, beta‐carotein, capric acid, lauric acid, myristic acid pentadecyclic acid, palmitic acid, palmitoleic acid, cis‐7 hexadecenoic, margaric acid, stearic acid, oleic acid, vaccenic acid, linoleic acid, linolenic acid, arachidonic acid, behenic acid, tricosylic acid, lignoceric acid	[129, 211]
*Aloe marlothii* A.Berger	GC‐MS	Carotenoids	Lutein, beta‐carotein vitamin c, capric acid lauric acid, myristic acid pentadecyclic acid, palmitic acid, palmitoleic acid, cis‐7 hexadecenoic, margaric acid, stearic acid, oleic acid, vaccenic acid, linoleic acid linolenic acid, arachidonic acid, behenic acid, tricosylic acid, lignoceric acid, fatty acids	[211]
*Amaranthus blitum* subsp. *oleraceus* (L.) Costea	GC‐MS	Phenol	Pyran based heterocycle, fatty acids, fatty ester, cyclododeca siloxane, alkyl siloxane, fatty acid, anyhydride	[220]
*Ansellia africana* Lindl.	Qualitative chemical assay	Saponin, alkaloids, tannins, terpenoids, steroids		[221]
*Artemisia afra* Jacq. ex Willd.	HPLC		Luteolin, quercetin, chlorogenic acid, neochlorogenic acid, scopoletin	[199]
*Asparagus africanus* Lam.	NMR, LC‐MS/Q‐TOF, Quantitative assay	Cardiac glycosides, proteins, flavonoids, tannins, saponins, steroids, terpenoids	Glucopyranoside, aspafricanol A, aspafricanol B, aspafricanol C, aspafricanol D, aspafricanol E, aspafricanene, aspafricanene A, acetylcaranine, 1,3,6,8‐naphthalenetetrol, stigmasterol, asparasaponin II, sarsasapogenin, prosopinine, glutinosone pandaroside C, cinncassiol C3	[217, 222, 223]
*Asparagus laricinus* Burch.	Qualitative assay	Glycosides, steroids, flavonoids, saponins, tannins, phlobatannins, terpenoids, reducing sugars		[175]
*Bulbine abyssinica* A. Rich.	Quantitative assay, qualitative assay	Tannins, phenolics, flavonoids, steroids, terpenoids, glycosides, saponins, alkaloids, proanthocyanins, condensed tannins		[176, 206]
*Burkea africana* Hook.	HPLC		Stigmasterol A, sitosterol B, catechin 4, glyceryl‐2‐stearate, glycerolmonostearate, epicatechin 5	[138]
*Cadaba aphylla* (Thunb) Wild	Qualitative assay, quantitative assay	Alkaloids, flavonoid, amino acids, proteins, anthraquinones, steroids, terpenoids, tannins, saponins, anthocyanins, coumarins, reducing sugars, oils and fats		[224]
*Cassia abbreviata* Oliv.	Qualitative assay, GC‐MS, HPLC‐PDA—MS/MS, NMR	Alkaloids, saponins tannins, phenols, flavonoids, terpenoids, sterols, phenols reducing sugars, total carbohydrates, cardiac glycosides, sterols, anthraquinone, coumarins, proanthosyanid, flavans	2,3‐Dihydro‐5‐hydroxy‐8‐methoxy‐2‐ (4‐methoxyphenyl) chromen‐4‐one; 3,4‐Dihydro‐2‐(4‐hydroxyphenyl)‐ 4‐methoxy‐2H‐chromen‐7‐ol	[154, 179, 225, 226]
*Cassine transvaalensis* (Burtt Davy) Codd Syn: *Elaeondedron transvaalense* (Burtt Davy) R.H. Archer	TLC chromatography Silica gel column chromatography	Triterpenoids	3 –Oxo‐28‐hydroxylbetuli‐20(29)‐ene, 3,28‐dihydroxylbetuli‐20(29)‐ene	[86, 218]
*Centella asiatica* (L.) Urb.	TLC, HPLC		Asiaticoside, Madecassoside, Asiatic acid	[227]
*Colophospermum mopane (*J. Kirk ex Benth.) J. Léonard	Quantitative assay, NMR	Tannins, saponins, flavonoids, cardiac glycosides, sterols and steroids, alkaloids, coumarins, diterpenes	Dihydrogrindelic acid, Dihydrogrindelaldehyde, methyl labd‐13‐en‐15‐oate	[172]
*Combretum hereroense* Schinz	UPLC‐MS	Triterpene		[155]
*Combretum imberbe* (Wawra)	TLC	Triterpene		[155]
*Diospyros lycioides* Desf.	TLC, quantitative analysis, qualitative analysis	Flavonoids, phenolics, tannins, terpenoids, steroids		[130]
*Drimia sanguinea* (Schinz.) Jessop	Quantitative assay, GC‐MS		Dotriacontane, benzothiazone, heptacosane, bumetrizole, phthalic acid (isomers), stigmasterol, hexanoic acid (derivatives), eicosanoic acid	[88]
*Elephantorrhiza elephantina* (Burch) Skeels	Quantitative assay, GC‐MS, qualitative assay	Alkaloids, flavonoid, amino acids, proteins, anthraquinones, steroids, terpenoids, tannins, saponins, anthocyanins, coumarins, reducing sugars, oils and fats	Dotriacontane, benzothiazone heptacosane, bumetrizole, phthalic acid (isomers), stigmasterol, hexanoic acid (derivatives), eicosanoic acid	[88, 228, 229]
*Englerophytum magalismontanum* (Sond.) T.D.Penn	Quantitative assay	Phenols		[228]
*Euclea undulata* Thunb.	NMR	Naphthoquinones	Epicatechin, 7‐Methyl‐juglone, α‐amyrin‐3O‐β‐(5‐hydroxy), ferulic acid	[230]
*Euphorbia regis‐jubae* (Webb & Berth)	Affinity and gel exclusion chromatography, quantitative assay	Catechin	Catechin, perulic acid, caffeic acid, vanilla acid, Rutin, Syringic acid	[231]
*Gomphocarpus fruticosus* (L.) W.T.Aiton	GC‐MS, TLC, Qualitative assay	Flavanols, phenols, terpenoids		[112, 184]
*Grewia flava* DC.	GC‐MS	Alkaloids, flavonoids, saponins, steroids, glycosides, anthraquinones, tannins	Tetradecanoic acid, hexadecanoic acid methyl ester, n‐hexadecanoic acid, chlorpyrifos, phytol, methyl stearate, octadecanoic acid eicosanoic acid methyl ester, disooctyl phthalate, tetratetracontane, tetracosanoic acid methyl ester, hentriacontane, octacosane, campesterol, stigmasterol, γ‐sitosterol, β‐amyrin, lupenone, lupeol, tarexerol	[185, 232]
*Harpagophytum procumbens* (Burch.) DC. Ex Meisn.	HPLC‐DAD, GC‐MS, HPLC	Phenolics, polyphenols, tannins flavonoids, flavanols	Rosmaric acid, harpagoside, arpagide	[233, 234]
*Helichrysum paronychioides* DC.	Quantitative analysis, GC‐MS		Dotriacontane, benzothiazone, heptacosane, bumetrizole, phthalic acid (isomers), stigmasterol, hexanoic acid (derivatives), eicosanoic acid	[88]
*Hypoxis hemerocallidea* Fisch., C.A. Mey. & Ave‐Lall.	Quantitative assay	Phenolics, tannins, gallotannins, flavonoids		[122]
*Ipomoea blongata* E. Mey. ex Choisy	Qualitative assay, quantitative assay	Cardiac glycosides, steroids, triterpenoids, alkaloids, flavonoids, tannins		[129]
*Malva neglecta* Wallr.	HPLC	Flavanoids, phenolic acids	Quercetin, kaempferol, gallic acid, chlorogenic acid, syringic acid, cinnamic acid	[212]
*Malvastrum coromandelianum* (L.) Garcke	NMR		Apigenin‐7‐О‐β‐6″ (p‐coumaroyl)‐glucopyranoside Apigenin‐8‐C‐glucopyranoside (vitexin)	[126]
*Moringa oleifera* Lam.	Qualitative assay, GC‐MS	Saponins, tannins, steroids, triterpenes, phlobatannins, flavonoids, glycosides, phytosterols	Brassicasterol, stigmasterol, campesterol, campesterol 2, 9,12‐octadecadienoic acid, gramisterol, Citrostadienol, beta‐sitosterol	[116, 235]
*Nicotiana tabacum* L.	Quantitative assay, GC ‐MS	Alkaloids, anthraquinones, cardiac glycosides, flavonoid, saponins, steroids, tannins, terpenoids	(S)‐Nicotine, 2‐methyl‐4, ‐acetoxy‐tetrahydropyran 5‐dihydrofuran, amitrole, citronellyl propionate, crotonaldehyde, isododecane, lavandulyl acetate, neophytadiene, pyridine, tetradecylaldehyde, trans‐phytol	[140, 159]
*Ozoroa paniculosa* var. *paniculosa* *Ozoroa paniculosa* var. *paniculosa * (Sond.) R.Fern. & A.Fern	Qualitative methods	Phenolics, condensed tannins, proanthocyanidins, gallotannin, flavonoids, flavanols		[190]
*Peltophorum africanum* Sond.	Qualitative and quantitative assays, TLC	Polyphenols, flavonoids, gallotannins, tannins		[203]
*Portulaca oleracea* L.	Qualitative and quantitative assays	Flavonoids, organic acid, polyphenols	Acetic acid, butyric acid, caffeic acid, chlorogenic acid, cinnamic acid, citric acid, coumaric acid, ferulic acid, formic acid, gallic acid, genistein, isoquercitrin, kaempferol, lactic acid, luteolin, malic acid oxalic acid, propionic acid, quercetin, rutin, succinic acid, tartaric acid, umbeliferone	[214]
*Rhoicissus tridentata* subsp*. Cuneifolia* *Rhoicissus tridentata* (L.f.) Wind & R.B.Drumm.	Qualitative and quantitative assays	Anthraquinone, coumarins, flavonoids, glycosides, phenols, phlobatanins, phytosterols resin, saponins, sterols and steroids, tannins, terpenoids, triterpenoids		[236]
*Rhus lancea* L. f. Syn: *Searsia lancea* (L.f.) F.A.Barkley	Quantitative assay	Tannins		[93]
*Ricinus communis* L.	GC‐MS, LC‐MS, NMR, Qualitative assay	Flavonoids, glycosides, phenolic compounds, tannins	1‐O‐α‐D‐glucopyranosyl‐1,2‐eicosandiol, 2:3‐Glc‐campesterol, 5α,6β‐dihydroxysitosterol, 6‐hydroxymethyllumazine 8‐methylcaffeine, All‐trans‐retinyl linolate, Bufotenine O‐glucoside, germanicol cinnamate, medicagol, pomordol, N‐demethyl‐ricinine, phylloquinone, phytenic acid, pubescenol, pubesenolide, ricinine, ricinoleic acid, sodium oleate	[235, 237]
*Ricinus communis* L.	Qualitative assay	Alkaloids, anthaquines cardiac glycosides, flavonoids, reduced sugars, steroids, tannins, terpenoids		[192]
*Schkuhria pinnata* (Lam.) Kuntze ex Thell.	Qualitative and quantitave assay	Alkaloids, cardiac glycosides, flavonoids, phlabotannins, saponins, steroids, tannins		[238]
*Sclerocarya birrea* Hochst.	Qualitative and quantitative assay	Alkaloids, flavonoids, glycosides, phenols, steroids, tannins		[201, 220]
*Securidaca longepedunculata* Fresen.	Qualitative analysis	Alkaloids, flavonoids, phenolic		[239]
*Senna italica* Mill.	Quantitative analysis, GC‐MS	Alkaloids, anthocyanins, anthraquinones, catechins, flavonoids, leucoanthocyanins, tannins	(y)‐Sitosterol, (α)‐Tocopherol‐β‐D‐mannoside, 1,2‐enzenedicarboxylic acid, mono (2‐ ethylheptyl) ester, 12‐docosenamide, 1‐heptacosanol, lupeol, n‐tetracontane, oxirane, phytol, squalene, stigmasterol	[240, 241]
*Senna tora* (L.) Roxb.	Quantitative assay, GC‐MS	Alkaloids, cardiac glycosides, flavonoids, reducing sugars, saponins, steroids, tannins, terpenoids	Carotene, cyclohexanedimethanol, hecamethyl cyclotrisiloxane, methyl stearate, neophytadiene, oxoalcohol, pentadecane, phenylethyl alcohol, pipecolic acid, tetradecane	[135, 236]
*Solanum incanum* L.	Qualitative assay, C‐NMR, DEPT, H‐NMR, IR, UV	Phenolic acid, saponins, tannins	Steroid derivative SIE2	[156, 235]
*Spirostachys africana* Sond.	GC‐ToF‐MS		n‐Hexadecanoic acid nonadecane,2‐methyl	[235]
*Tarchonanthus camphoratus* L.	Qualitative assays	Flavonoids, phenolic compounds, saponins, steroids tannins, terpenoid		[194]
*Terminalia sericea* Burch. Ex DC	UPLC‐MS	Triterpene		[155]
*Thamnosma* rhodesica (Baker f.) Mendonça	3D NMR, C‐NMR, H‐NMR	Coumarins, furanocoumarins	1‐hydroxy‐10‐methylacridone, byakangelicin, cnidili, gravacridonediol, imperatorin isopimpinellin, marmesin, rhodesiacridone, rutacridone, xanthotoxin	[205]
*Tribulus terrestris* L.	HPLC‐MS	Saponins	Astragaloside, hypericin, isomer of quercetin 3‐O‐arabinosyl galactoside, isoquercitrin, kaempferol 3‐ gentiobioside, microcephalin I, quercetin 3‐gentiobioside, quercetin‐3‐O‐(2,6‐α‐L‐dirhamnopyranosyl‐β‐D‐glucopyranoside), terrestrinin G, terrestrinin T, terrestrosin F, terrestrosin G, terrestrosin I, tribufuroside J, tribulosin, tribuluside A	[153]
*Urginea sanguinea* Schinz	C‐NMR, EL‐MS, FAB‐MS, H‐NMR		3‐Hydroxy‐4‐methylbenzoic acid, 5a‐4,5‐dihydroscillaren A, n‐butanol fr, Phioroglucinol, Phloroglucinol, 1‐B‐D‐glucopyranoside (phiorin), Salicylic acid, Scillaren A, stigmasterol	[242]
*Vachellia karroo* (Hayne) Banfi & Galasso	Quantitative Colometric phenolic assays	Phenolics		[243]
*Vachellia karroo (*Hayne) Banfi & Galasso	GC‐MS	Phenolics		[195]
*Vachellia karroo* (Hayne) Banfi & Galasso	TLC, H NMR, UHPLC‐qTOF‐MS		Baicalein, catechin, epicatechin epigallocatechin, kaempferol rutinoside, methyl gallate, myricetin rutinoside, quercetin, quercetin rutinoside, rutin	[121]
*Vachellia nilotica* (L.) P.J.H.Hurter & Mabb		Anthraquinone, flavonoids, saponins, tannins		[244]
*Vachellia tortilis* (Forssk.) Galasso & Banfi	Qualitative analysis, H NMR, HP‐TLC, TLC, UHPLC‐qTOF‐MS	Alkaloid salts polyphenols, alkaloids, carotenoids, flavonoid, saponins, phenolic acids, phenols, saponin, sterols, tannins, triterpene, volatile oils	Baicalein, chrysoeriol glucopyranoside, cyanidin rhamnoside, dihydroacacipetalin, kaempferol, kaempferol rutinoside, luteolin glucoside, myricetin rutinoside	[120, 121]
*Withania somnifera* (L.) Dunal	HPLC	Phenols	Withanolodine A, 12‐seoxywithastramonolide, Withanoside‐IV, Withaferin A Withanolide A, Withanone Physalgulin‐D, Withastromonolide, 28‐hydroxywithanone	[119, 245, 246]
*Ximenia americana* L. *var. microphylla* Welwe. Ex Oliv.	Quantitative assay, FTIR, HP‐TLC, HP‐LC, GC‐MS	Alkaloids, fatty acids, flavonoids, glycosides, phenolics, phytosterols, saponins, tannins, terpenoids	2,6,10,14‐Tetramethyloctadecane, chloracetamide‐n‐methanol, diethylhexylphthalate, elaidic acid methyl ester, nonadecane, oleic acid methyl ester, palmitic acid methyl ester, stearic acid methyl ester, ximeninic acid	[119, 247]
*Ziziphus oxyphylla* Edgew Syn*: Ziziphus acumi nata* Royle	H‐ NMR, EI‐MS	Flavonoids, phenolic acids	Caffeic acid, catechin, ferulic acid, quercetin	[247]

Abbreviations: CC, column chromatography; DEPT, distortionless enhancement by polarisation transfer; EI‐MS, electron ionisation‐mass spectrometry; FTIR, Fourier transform infrared; GC‐MS, gas chromatography‐mass spectrometry; HPLC, high performance liquid chromatography; IR, infrared; LC‐MS, liquid chromatography‐mass spectrometry; NMR, nuclear magnetic resonance; TLC, thin layer chromatography; UHPLC‐qTOF‐MA, ultra‐high performance liquid chromatography‐quadrupole time‐of‐flight mass spectrometer; UPLC, ultra performance liquid chromatography; UV, ultraviolet.

Extraction of bioactive compounds in different plant parts is highly dependent on the extraction solvents. The solvent is selected based on the polarity of targeted compounds, purpose of extraction, cost of solvent, safety of solvent, and potential energy demand for heating solvents [74, 75]. The common use of water as an extractant can be attributed to its general use in traditional medicine to mimic ethnoveterinary practices involving maceration, decoction, poultice and infusions [76]. In the reviewed data (Figure 6), the use of water as an extractant was reported in 16% of the studies with methanol being the most prominent solvent used in majority of the studies (22%). Compounds with antimicrobial activity such as ketones, aldehydes and organic acids are extracted in high quantities using methanol as a solvent [77]. Ethanol (alcohol) is also widely used in traditional medicine as an alternative extractant to water in the reviewed literature and its use was calculated at 14%. Solvents reportedly used for plant extractions that had a low rate of use included petroleum ether (4%) and chloroform (4%) (Figure 4). The type of solvent used in plant extraction is also associated with the experimental analysis that is conducted, including biological activity, safety, phytochemical quantification, and bioactive compound isolation. Generally, biological activity (in vitro studies) in medicinal plants is investigated based on the ethnobotanical practices recorded by indigenous communities.

In the current review, the ethnoveterinary practices of medicinal plants used by the Batswana communities were assessed by examining the medicinal properties e.g., antimicrobial, antiparasitic, anti‐inflammatory, antioxidant, and toxicity of the plants. As a common trend in medicinal plant research, in vitro biological assays conducted accounted for 84% of the studies with in vivo assays contributing towards 16% of the research. Basically, plants that had noteworthy in vitro activity and have potential in drug discovery (interesting bioactive compounds) are further analysed in vivo, and such tests produce more accurate results than in vitro experiments. Unfavourable in vitro results may eliminate the need for further in vivo studies [78].

##### 3.3.1.1 Antimicrobial Activity

**FIGURE 7:**
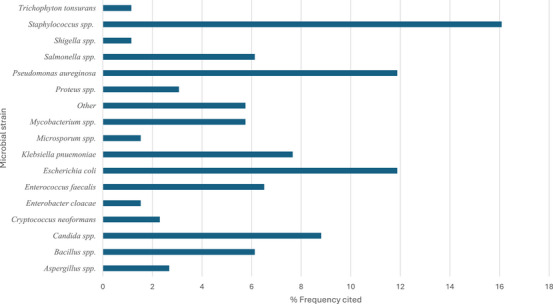
Microbial strains frequently used for determining the antimicrobial effects of plants with ethnoveterinary records among the Batswana in southern Africa. `Others: *Moraxella cattarhalis*, *Micrococcus* sp., *Listeria monocytogenes*, *Aeromonas hydrophila*, *Acinobacter calcoaceticus*, *Yersinia enterocolitica*, *Fusarium oxysporum*, *Mycoplasma hominis*, *Haemophilus influenza*, *Sporothrix schenckii*, *Citrobacter* spp., *Serattia marcescens*

Antimicrobial screening is the most common method applied for primary analysis of biological activity because it is quick, efficient and the resources required are usually readily affordable. Particularly, the serial dilution microplate assay is the most preferred method for evaluating the minimum inhibitory concentration (MIC) of plants against different test microbial strains, with more than 2 900 citations to date (accessed 08/02/2025) [79]. Following the recommended classification [80, 81], extracts with MIC values > 0.32 mg/mL are regarded as exerting weak antimicrobial activity. However, higher MIC values may still be relevant for ethnopharmacological investigations [80]. The criteria for establishing activity of plant extracts are variable across difference sources, with noteworthy MIC values ranging from 8000 to ≤ 160 µg/mL [81]. MIC values < 0.1 mg/mL are also regarded as good by another author, while MIC values > 0.625 mg/mL was considered as weak [82, 83]. For plant extracts used in traditional medicine, the MIC values of below 8 mg/mL are considered active [84]. Other authors considered a value as high as 12.5 mg/mL as active [85]. However, this review considers MIC as good/noteworthy when the activity is from <0.02 to 0.16 mg/mL [80]. Additionally, other in vitro assays used to determine antimicrobial activity included TLC bioautography, and agar disc diffusion [86, 87, 88]. Based on the results reported in Table 4, the serial dilution method was the most frequently applied technique followed by the agar disc diffusion assay. During the antimicrobial bioassays, microbial and fungal strains associated with animal gastrointestinal illnesses, skin infection, mastitis, and respiratory diseases amongst others were investigated in several studies, possibly due to the medicinal utilisation of the plants in ethnoveterinary medicine. The five most studied strains were *Staphylococcus* spp. (16.1%), *Pseudomonas aeruginosa* (11.9%), *Escherichia coli* (11.9%), *Candida spp*. (8.8%) and *Klebsiella spp*. (7.7%) (Figure 7). These pathogens are associated with most of the commonly reported ailments treated with medicinal plants. *Aloe marlothii* and *Aloe zebrina* extracts had excellent activity (MIC = 0.028 and 0.039 mg/mL) against *E. coli* [89, 90]. This corroborates the perceived efficacy of the two *Aloe* species by the communities as they are reportedly used for diarrhoea and other infections [64, 91]. *Cassia abbreviata* extracts had noteworthy activity against *P.aeruginosa* (MIC = 46.88 µg/mL), *K. pneumoniae* (MIC = 46.88 µg/mL), and good activity against *C*. *albicans* (MIC = 93.75 µg/mL) [92]. *Dicerocaryum eriocarpum* had good activity against *Mycobacterium aurum* (MIC = 0.156 mg/mL) but weak activity against *C*. *albicans* (MIC = 4.5 mg/mL) [90]. None of the conditions reported to be treated by *D. eriocarpum* are related to the tested pathogens [52, 62, 63, 91], therefore the results do not support the traditional use. *Searsia lancea* had excellent activity (MIC range = 0.02–0.08 mg/mL) against the mastitis‐causing pathogens *E. coli, Streptococcus agalactiae, Streptococcus dysgalactiae* and *Streptococcus uberis* [93]. This may merit the use of *S*. *lancea* for treating diarrhoea resulting from one or more of the tested pathogens [91].

Diverse techniques are applied when observing the in vivo activity of medicinal plant extracts and these include visual examination of wound healing activity after topical application [94], faecal bacterial load [95], and determining secondary infection symptoms such as pulmonary burden after ingestion [96]. Study by Amoussa et al. [95] revealed that *A*. *afra* had notable in vitro activity (MIC range = 0.25–0.312 mg/mL) against non‐typhoidal *Salmonella* field isolates and ATCC strains and demonstrated the efficacy of the plant extracts against biofilm formation. From the same study, there is evidence of in vivo activity as the orally administered *A. afra* at 200 mg/kg/bw extracts completely eliminated the faecal bacterial load in mice after eight days [95]. *Terminalia sericea* was effective at reducing the induced wound associated with *S. schenckii* infection on mice, after excellent activity (MIC = 0.03 mg/mL) against *S*. *schenckii* was reported [97]. In vivo studies can also reveal additional information about the mode of action of the plant extract [96]. Additionally, these studies also confirm the potential of medicinal plant extracts to function as multitarget drugs [98]. A combination of in vivo and in vitro studies is important to further determine the efficacy of the plant extracts against the target strains. *Artemisia afra* is one plant that has been reported to have good antimicrobial activity in vitro against several strains of *Salmonella* spp. which is also supported by noteworthy in vivo activity [95]. However, an in vivo study revealed that the orally administered *A. afra* leaf water and DCM extracts had no antimycobacterial activity, despite the positive in vitro activity [96]. The phytochemicals associated with antimicrobial activities are terpenoids and phenolic compounds with hydroxyl groups which interrupt the bacterial cell membrane permeability [99]. The presence of such compounds in the aqueous bark extract of *Ximenia americana* [100] is consistent with the good antimicrobial activity reported against *Bacillus subtilis* (MIC = 0.20 mg/mL). It is presumed that the mode of action of most antimicrobial agents is by creating structural changes to the bacterial cell membrane which affects the integrity of the membrane [101]. Commonly, this results in lysis of the cells and leakage of the contents leading to cell death [98, 101].

##### 3.3.1.2 Antiparasitic Activity

Determining the antiparasitic activity of medicinal plants is of great significance in subtropical regions because the climatic conditions are breeding grounds for parasite infestations especially in animals [102]. In Batswana ethnoveterinary, examples of plants with notable *in vitro* antiparasitic activity includew *Acokanthera oppositifolia, Aloe ferox, Aloe marlothii, Artemesia afra, Combretum hereroense, Dicerocaryum eriocarpum, Elephantorrhiza elephantina, Nicotiana tabacum, Sclerocarya birrea, Securidaca longepedunculata, Thamsosma rhodesica*, and *Ximenia americana* (Table 5). In vitro assays such as egg hatch and parasite mortality assays are important in determining plant pesticidal activity [103]. However, in vitro studies usually target a single parasite, meanwhile multi‐parasitism is common in livestock [104]. On the other hand, in vivo assays typically involve topical application of plant extracts for external parasites, or oral ingestion for effective treatment of gastrointestinal parasites [105]. Parameters used to determine antiparasitic activity can reveal secondary effects, making them a suitable multitarget approach [106]. In vitro anthelminthic activity of *Aloe ferox* was reported [107] and in vivo studies supporting the activity of the extracts were conducted [54, 108]. The authors observed that water extracts administered orally were able to reduce worm eggs and inhibit larval development [54, 108]. As shown in Table 2, *Nicotiana tabacum* was cited twice in this report for use against parasites [63, 109]. It has been tested against several parasites yielding positive results for in vitro and in vivo [110]. *Ximenia americana* had good activity (100% mortality) against *Rhipicephalus microplus*, however it has been reportedly used for internal parasites [91]. Fatty acids and fatty acid esters are believed to be responsible for nematocidal activity exhibited by plant extracts [111]. Phenolic compounds which are usually produced by plants to deter pests, also contribute to antiparasitic activity of plants [112]. Phytochemicals with proven nematocidal activity (e.g., tannins), have been isolated from plants used by the Batswana, including ganglion stimulants from *Nicotiana tabacum* leaves, which is similar to the active ingredient of levamisole (control agent) [113].

##### 3.3.1.3 Anti‐Inflammatory Activity

Inflammation can be a result of physical injury, infection or other ailments [114, 115]. In vitro activity is commonly determined by the inhibition of cyclooxygenase and lipoxygenase enzymes [116]. Anti‐inflammatory and other pain‐related assays had the highest rate (47%) of in vivo studies reported (Table 6). Physical evidence of pain and inflammation can be assessed visually in the tail‐flick assay and paw lick test [117], and by observing morphological changes during wound healing [118]. In vivo studies on *Vachellia tortilis* seed extracts exhibited noteworthy anti‐inflammatory response in mice with comparable activity (reaction time of 6.55 sec) in the group treated with diclofenac sodium (reaction time of 6.58 sec) and morphine sulfate (reaction time of 12.33 sec) [117]. The chemical profile of *Vachellia tortilis* revealed interesting phytochemicals such as myricetin rutinoside and luteolin glucoside which may be responsible for providing pain relief [119, 120, 121]. This supports the efficacy of *V. tortilis* for its traditional use of treating diarrhoea [91]. Decoction of *Acokanthera oppositifolia* leaves is used to expel parasites [53]. The extracts have excellent COX‐1 inhibition and COX‐2 inhibition activity, indicating good anti‐inflammatory activity with good in vitro nematocidal activity [122]. The anti‐inflammatory activity may be related to the presence of compounds which give the plant good antioxidant efficacy [123, 124]. *Vachellia karoo* extracts had good anti‐inflammatory activity in mice which lasted up to 2 days after the treatment application. The 100 mg/kg dose produced 1.81 % inhibition, and the 200 mg/kg dose had 1.07% inhibition of inflammation, which is comparable to the inhibition of the positive control agent (1.38 %) used, indomethacin at 10 mg/kg [123]. The results support the use of *V*. *karroo* to treat fractures and skin diseases [64, 76]

##### 3.3.1.4 Antioxidant Activity

Plants with antioxidant activity are potential candidates for preventing certain inflammatory diseases [124]. Evidence of the antioxidant effect of the ethnoveterinary plants used by the Batswana have been assessed (Table 7). For instance, *A. oppositifolia*, which contains flavonoids, proanthocyanidins and quercetin equivalents did not exhibit in vitro NO‐ radical inhibition, but was active against ABTS free radicals, DPPH free radicals, hydroxyl free radical, superoxide anion scavenging activity and iron reducing activity [17, 125, 126].

In terms of the ethnoveterinary records, snake bites are one of the most common conditions treated by plants among the Batswana of southern Africa (Table 2). As the toxicity effect of snake venom is associated with inducing oxidative stress and inflammatory response [127, 128], establishing the efficacy of medicinal plants used to treat snake bites becomes important. Some plants assessed for antioxidant activity are used to treat snake bite, with varying antioxidant activity reported. *Aloe ferox* extract exhibited notable DPPH (60%) and ABTS (80%) scavenging activity at the highest tested concentration of 0.4 mg/mL [129]. The DPPH scavenging activity of *Diospyros lycioides* was assessed using TLC and there were several bands in chromatograms eluted using three different systems indicating the presence of polar, non‐polar and neutral polarity with some antioxidant activity [130]. *Moringa oleifera* is reportedly used for cough and other unspecified conditions [63, 131]. The extracts of *M*. *oleifera* had noteworthy in vitro DPPH, OH and NADH scavenging activity as well as in vivo ferrous ion chelating activity [132].

#### 3.3.2 Toxicity and Safety of Medicinal Plants With Ethnoveterinary Records

Toxicity bioassays are necessary to determine the safety of herbal remedies in animals and humans [88]. The selectivity index (cytotoxicity) of plant extracts is important pecially in targeting specific cancer cell lines [133]. Cytotoxicity studies need to be conducted in parallel with the biological assays to confirm the efficacy for the plants or its general toxicity [134]. Some of the plants with ethnoveterinary records among the Batswana in southern Africa have been assessed for their safety with diverse responses (Table 8). As with other biological assays, in vitro cytotoxicity results will not always have the same results as acute toxicity tests. For instance, *Senna tora* leaves (ethyl acetate extracts) were reported to have moderate cytotoxicity (LC_50_ = 35.246 µg/mL) when using the brine shrimp assay, while no acute oral toxicity (LD_50_ = 4263.906 mg/kg b.w.) was observed in an in vivo assay using mammalian subjects [135].

Biomarkers such as glucose, urea, creatinine and alkaline phosphates are used to determine in vivo toxicity. These biomarkers usually indicate the degradation of vital organs. The impact on renal function, which is assessed by evaluating serum ureal and creatine concentrations in mice models is another indicator of the toxicity of *Senna italica* extracts [136]. In vivo assays account for 35% of the toxicity and safety assays reported (Figure 6). Some parameters used to determine acute oral toxicity are signs of hepatic damage [137], sedative or paralytic effect [138], loss of body weight [139], and mortality [94, 113, 140] reported on the subacute toxicity effect after topical application of plant extract. Mice treated *with Nicotiana tabacum* extract in a trial for activity against ticks (*Rhipicephalus microplus*) exhibited no signs of toxicity (i.e., skin reaction, intestinal distress, mortality) after 3 weeks [140]. The results indicate the efficacy and safety of *N. tabacum* for external parasites, leaving opportunity for investigating the efficacy and safety in terms of its traditional use in treating internal parasites [63, 109, 131]. The in vivo toxicity study revealing the safety of topically applied *Terminalia sericea* was part of a study investigating in vivo anti‐inflammatory activity [94]. This further supports the topical application of *T*. *sericea* (Table 2).

#### 3.3.3 Profiling, Quantification and Identified Phytochemicals of Plants With Ethnoveterinary Records Among the Batswana of Southern Africa

**FIGURE 8:**
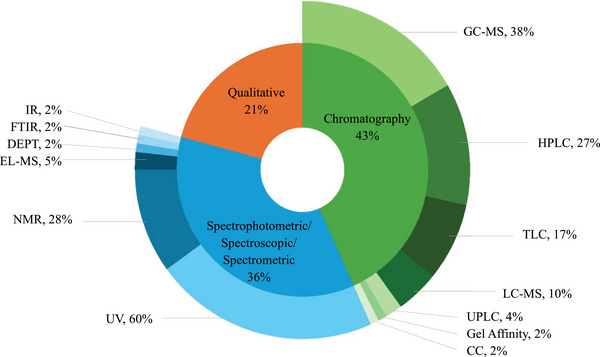
Overview of the applied methods for phytochemical analysis of plants with ethnoveterinary records among the Batswana in southern Africa. Qualitative methods include colorimetric methods used to detect the presence of metabolites. IR = Infrared, NMR‐ Nuclear magnetic resonance, DEPT = Distortionless enhancement by polarisation transfer, EL‐MS = Electron ionisation mass spectrometry, FTIR = Fourier transform infrared, UV = ultraviolet, HPLC = high performance liquid chromatography, TLC—Thin layer chromatography, GC‐MS = Gas chromatography‐mass spectrometry, LC‐MS = Liquid chromatography‐mass spectrometry, UPLC = Ultra performance liquid chromatography, CC = Column chromatography

Secondary metabolite production in plants is regulated by a range of factors, including biotic and abiotic stresses, physiological and developmental processes, chemical and mechanical elicitors, and cultivation and post‐harvest conditions. Despite the diversity of factors influencing their synthesis, plant secondary metabolism is predominantly directed towards the production of phenolics (≈45%), followed by terpenoids and steroids (≈27%), alkaloids (≈18%), and other compound classes (≈10%) [141]. From the current findings, a significant portion (51.72%) of the 116 plants with ethnoveterinary records have been profiled to establish their phytochemicals (Table 9). Phytochemical profiles varied among the identified species, with phenolics constituting the most abundant group, while terpenoids, steroids, and alkaloids were also present across the plant species. Generally, phytochemical analysis methods employed are chosen based on type of compounds sought, metabolites of interest, or the cost of analysis [142, 143]. Chromatography methods are the most reported techniques used for phytochemical analysis (Figure 8). Earlier studies on phytochemical profiling primarily employed qualitative methods, such as colorimetric assays, which confirms the presence of phytochemical groups through precipitation, formation of foam or colour change in solution [144]. This method is usually applied at preliminary screening stages, as it provides a simple, quick and cost effective way of detecting phytochemicals without the need for sophisticated instrumentation [145].

Gas chromatography is a relatively low cost method for identifying volatile organic compounds [146] and is the most commonly cited chromatography method due to volatility of phytochemicals such as terpenoids [147]. Ultraviolet light spectrophotometry is used to quantify phytochemicals that were detected using qualitative methods such as Sakowski test for steroids, Lieberman–Uchard's test for triterpenes, Wanger's test for alkaloid, ferric cyanide test for phenolic compounds and ferric chloride test for flavonoids [148].The phenolic compounds and flavonoids identified from *Ximenia americana* using the Folin–Ciocalteu and the Adom and Liu methods, respectively, have a wide range of biological activities [149]. Phenolic compound exhibit in vitro anthelmintic activity [150, 151], which may contribute to the reported use to treat internal parasites (Table 2).

Chromatographic methods are used for separating, detection and quantification [152].Ultra‐performance liquid chromatography analysis of *T. sericea* extracts detected saponins which exhibited in vitro anti‐inflammatory activity on LPS treated RAW 264.7 cells [153]. The leaves, stem, roots and bark of *T. sericea* are used for several conditions including cough, internal parasites, diarrhoea and retained placenta (Table 2). The methanol extracts have in vitro anti‐microbial activity against *Staphylococcus aureus, Bacillus cereus, Staphylococcus epidermis, Enterococcus faecalis, Escherichia coli, Salmonella typhirium, Pseudomonas aureginosa* and *Klebsiella pneumonia* (average MIC ≥ 1.49 mg/mL), and the acetone and methanol extracts have in vivo *w*ound healing activity observed, indicative of in vitro anti‐inflammatory activity (2 g/10 g dose) [97].

Spectrophotometric methods detect phytochemicals by detecting the functional groups present in a compound or the presence of conjugation within a compound [152]. The ─OH functional groups containing compounds identified from *Cassia abbreviata*, was identified as a flavan which was named 2,3‐dihydro‐5‐hydroxy‐8‐methoxy‐2‐(‐4‐methoxyphenyl)chromen‐4‐one [154]. The compound did not exhibit greater antiplasmodial activity (IC_50_ = 26.02 µg/mL) than the crude extract (IC_50_ = 13.31 µg/mL), or positive controls used, Chloroquine (IC_50_ = 0.026 µg/mL), mefloquine (IC_50_ = 0.03 µg/mL) and quinine (IC_50_ = 0.09 µg/mL) [154]. Despite the unspecified ethnoveterinary use of *C. abbreviata* (Table 2), flavans and other flavonoids exerted antioxidant, anti‐inflammatory and antimicrobial activities which contribute to treating several conditions [155, 156].

The phytochemicals have a wide range of therapeutic properties, and the presence and concentration of specific phytochemicals can predict the bioactivity. The antioxidant activity of *Ximenia afra* leaf extracts can be attributed to the presence of procyanidins, and quercetin, compounds found to contribute to antioxidant activity [157]. The phytochemicals associated with antimicrobial activities are terpenoids and phenolic compounds with hydroxyl groups which interrupt the bacterial cell membrane permeability [99]. Therefore the presence of phenolic compounds and terpenoids (alkaloids, fatty acids, flavonoids, glycosides, phenolics, phytosterols, saponins, and tannins) in the aqueous bark extract of *Ximenia americana* [100] is consistent with the good antimicrobial activity reported [158].

The extensive chemical profiling for *A*. *tortilis* [121] revealed the presence of phytochemicals such as myricetin rutinoside and luteolin glucoside which may provide pain relief [119, 120, 121]. Fatty acid and fatty acid esters are believed to contribute to nematocidal activity of plant extracts [111]. Tannins and phenolic compounds are usually produced by plants for defense in response to biological attack [112, 113]. This is evident in *Nicotiana tabacum* which has a high phenolic compound content [159] and has shown good antiparasitic activity. Qualitative analysis of *Opuntia ficus‐indica* extract, which has proven in vivo anthelmintic activity [160], has revealed the presence of polyphenols and phenolic compounds [161].

### 3.4 Clinical Trials of Plants With Ethnoveterinary Records

Clinical trials are required to determine long term toxicity, side effects and drug interactions and possible secondary benefits of phytotherapies [162, 163, 164]. A significant challenge in veterinary clinical trials is overcoming bias [165], which is compounded by the existing obstacles associated with clinical trials for phytotherapies. The complex nature of medicinal plants used in phytotherapies can cause inconsistencies and variations in phytochemical concentrations [166], therefore standardisation of protocols can be difficult [167]. The placebos used in phytotherapy clinical trials often pose an additional challenge as they fail to have the same colour, texture and smell, effectively not being an analogous compound. This brings to question the validity of the trial as a double‐blind study [168]. These challenges and more can limit the credibility of trial results, creating obstacles in regulating the industry [169]. Despite this, the clinical trial registry continues to be updated with new medicinal plant remedies in veterinary medicine (https://veterinaryclinicaltrials.org/studies/?term~recruiting_status = R, access 20/11/2024). A randomised, placebo‐controlled double‐blind clinical trial conducted on *Cannabis sativa* oil was reported to be beneficial in managing chronic pain by reducing inflammation and oxidative stress in canines [170]. There have been no reports to date of veterinary clinical trials for medicinal plants used by the Batswana people.

## 4 Conclusions

This review underscores the critical importance of traditional livestock husbandry practices among Batswana communities in southern Africa, where plant‐based remedies serve as essential tools for managing livestock health. The appraisal on ethnoveterinary knowledge uncovers the diversity of plants that the Batswana people use to treat a range of conditions such as parasitic infestations, wounds, infectious diseases and complications from animal bites. In the context of limited access to conventional veterinary care, these culturally rooted practices demonstrate the ingenuity and adaptability of indigenous knowledge systems in addressing diverse livestock health challenges. The continued reliance on these practices highlights their relevance not only as a means of sustaining livestock productivity but as a reflection of the deep connection between cultural heritage and indigenous medicinal plants. A comprehensive inventory of 116 plant species from 44 families was recorded, showcasing the remarkable biodiversity leveraged in treating nine major categories of livestock health conditions. Commonly used plant parts, such as roots and leaves, were prepared through methods such as decoctions and infusions, with oral and topical routes of administration being predominant. The presence of phytochemicals and the biological activities of the plants support the therapeutic potential of the plants. This provides empirical evidence to support ethnoveterinary knowledge. However, the presence of phytochemicals cannot be used as a sole determinant or factor for biological activity validation as other factors such as bioavailability can influence the efficacy and potency of plant extracts in vivo. Complementary in vivo studies and clinical trials can provide essential information on safety, pharmacological effect, and the correct dosage of ethnoveterinary medications, supporting the adoption of herbal remedies and their integration into modern veterinary health practices. Overall, the ethnoveterinary practices of the Batswana are of great benefit to underserved communities. The incorporation of plant‐based ethnoveterinary remedies into primary animal healthcare may be advantageous for rural communities given their dependence on livestock for survival.

## Author Contributions

AOA and NAM conceptualised the study and edited the manuscript, TGM, NS and MVC sourced for the literature, conducted formal analysis and involved in the initial draft. JAA, SOA and LJM provided critical insight, edited the manuscript, and are involved in the supervision of the project. NAM, SOA and AOA secured the fundings for the project. All authors have read and agreed to the final version of the manuscript.

## Funding

This work is based on the research supported by the South African National Department of Agriculture (DoA). A.O.A. received funding from the South African Research Chairs Initiative of the Department of Science, Technology and Innovation (DSTI)‐National Research Foundation (NRF) of South Africa (Grant No: RCHDI2411105279212). The financial support provided by the Higher Degree Committee of the Faculty of Natural and Agricultural Sciences (FNAS), North‐West University to TGM is sincerely appreciated. The opinions, findings, and conclusions or recommendations expressed are those of the authors alone; DoA and NRF accept no liability whatsoever in this regard.

## Conflicts of Interest

The authors declare no conflict of interest.

## Institutional Review Board Statement

This study was approved with reference number NWU‐01409‐23‐A9 by the Faculty of Natural and Agricultural Sciences Research Ethics Committee (FNASREC), North‐West University, South Africa.

## Supporting information




**Supporting File 1**: cbdv71121‐sup‐0001‐SuppMat.docx

## Data Availability

Data used for this study have been included as part of the article.
