# Supplementary material for: Alternative Approaches and Plant‐Based Remedies for Livestock Health Management Among the Batswana of Southern Africa: A Review
Source: Chem Biodivers. 2026 Apr 17;23:e03248. doi: 10.1002/cbdv.202503248 (PMC13090005; doi:10.1002/cbdv.202503248)
Supplement: Supplementary file 1 — Supporting File 1: cbdv71121‐sup‐0001‐SuppMat.docx [file CBDV-23-e03248-s001.docx]

**Supplementary Materials**

**Table S1:** Overview of the plant parts utilised for managing livestock health conditions among the Batswana people in southern Africa.

| **Plant parts** | **Frequency** | **Percentage (%)** |
| --- | --- | --- |
| **Main parts** | | |
| Root | 99 | 33.2 |
| Leaves | 78 | 26.2 |
| Whole plant | 36 | 12.1 |
| Bark | 22 | 7.38 |
| **Unspecified** | 18 | 6.04 |
| **Other parts = 15.08%** | | |
| Bulb | 11 | 3.69 |
| Flower | 6 | 2.01 |
| Fruit | 6 | 2.01 |
| Stem | 6 | 2.01 |
| Tuber | 5 | 1.68 |
| Aerial parts | 3 | 1.01 |
| Branch | 3 | 1.01 |
| corms | 2 | 0.67 |
| nods | 1 | 0.34 |
| Rhizome | 1 | 0.34 |
| Seeds | 1 | 0.34 |
| **TOTAL** | **298** | **100** |

**Table S2:** Overview of the methods of preparing plants utilised for managing livestock health conditions among the Batswana people in southern Africa.

| **Preparation** | **Frequency** | **Percentage (%)** |
| --- | --- | --- |
| Unspecified | 95 | 35.98 |
| Decoction | 63 | 23.86 |
| Infusion | 42 | 15.91 |
| Poultice | 27 | 10.23 |
| Ground | 26 | 9.85 |
| Other methods of preparation reported | | |
| Maceration | 9 | 3.41 |
| Burn | 1 | 0.38 |
| Roosted | 1 | 0.38 |
| **TOTAL** | **264** | **100** |
